# UV Spectrometric Indirect Analysis of Brominated MWCNTs with UV Active Thiols and an Alkene—Reaction Kinetics, Quantification and Differentiation of Adsorbed Bromine and Oxygen

**DOI:** 10.3390/ma6083035

**Published:** 2013-07-24

**Authors:** Sven Hanelt, Jörg F. Friedrich, Asmus Meyer-Plath

**Affiliations:** Federal Institute for Materials Research and Testing (BAM), Division 6.6, Unter den Eichen 87, Berlin 12200, Germany; E-Mails: joerg.friedrich@bam.de (J.F.F.); asmus.meyer-plath@bam.de (A.M.-P.)

**Keywords:** carbon nanotubes, CNTs, UV-spectrometry, bromination, thiols, mercaptans, disulfides, quantification, kinetics

## Abstract

Indirect UV-absorption spectrometry was shown to be a valuable tool for chemical characterization of functionalized carbon nanotubes (CNTs). It complements data from X-ray photoelectron spectroscopy (XPS) or FTIR analysis since it helps to clarify the type and concentration of functional groups. The principles of indirect application of UV-spectrometry and its mathematical interpretation are discussed. Their facile application, together with their adequate sensitivity and high flexibility, make UV-absorption-based approaches a valuable alternative to fluorescence spectrometry. Here, the approach was applied to the chemical analysis of oxidizing substances on CNTs. For this, pristine CNTs of low but finite oxygen content as well as brominated CNTs were analyzed by reaction in suspension with UV-active thiol reagents and a styrene derivative. It was shown that carefully selected reagents allow differentiation and quantification of bromine and generally oxidizing entities like oxygen. For brominated CNTs, it was shown that physisorbed bromine may dominate the overall bromine content.

## 1. Introduction

In analytical chemistry, ultraviolet-absorption spectroscopy (UV spectroscopy) is applied for determining the concentration of a UV-active compound in the gas phase or in solution. It is a standard technique to quantify dissolved analyzes. In the research field of carbon nanotubes, functionalized CNTs are characterized with respect to their chemical character by techniques like potentiometric titration [1] and derivatization with group-specific reagents that contain marker atoms like fluorine. This allows the quantification of chemically derivatized reactive entities on CNTs with appropriate elemental analytical techniques. Additional bond polarity differentiation capabilities of techniques like X-ray photoelectron spectroscopy (XPS) or solid state nuclear magnetic resonance spectroscopy (NMR) may help to verify the bond state of the reagent or to use marker-free reagents. The selectivity and yield of derivatization reactions used for labeling is an issue that has to be addressed to assess the reliability of the quantification result.

Fluorescence spectroscopy is a well-known technique and has been used for the detection and quantification of surface functional groups on carbon materials after derivatizations with group-selective labeling reagents like, for example, anthracene or dansyl derivatives [2]. Usually, the “depletion” approach is performed. It is an indirect method to quantify reactive functional groups on solid samples by monitoring the depletion of the concentration of the reagent in solution by coupling reactions with functional units on the solid surface [3]. Pellenbarg* et al*. described a sampling technique to monitor the depletion similar to the one presented here by taking aliquots of a solution containing dissolved analyte and parts of the solid sample [4].

Quantification of functional groups and entities on solid samples like carbon nanotubes by fluorescence spectroscopy has been studied before. Although it is in principle more sensitive and has a lower detection limit than the UV-spectroscopic approach describe here, it is the intention in this work to point out the usefulness, preciseness, and flexibility of UV-spectrometry as an alternative procedure. UV-spectroscopy, not requiring fluorescent but UV-radiation-absorbing reagents, offers a broader choice of reagents and may therefore be applied more frequently, for instance not only in depletion but also in “enrichment” studies, where a UV-absorbing substance desorbs from the surface into the solvent or forms in the solvent in reactions with desorbed species. In addition, hetoroatom-labeled UV-active reagents may be designed and synthesized with more ease and flexibility than fluorescent reagents. This way, depletion studies with labeled reagents can be coupled with subsequent elemental analysis of CNTs that immobilized the reagent by coupling reactions, for instance by XPS-analysis after washing and drying of the labeled CNT sample.

UV-Vis-(NIR)-spectroscopy has also been considered as an analytical tool for the determination of the overall CNT content and the quality of purification procedures [5]. A more recent publication shows how to quantify highly purified single-walled CNTs (SWCNTs) in water containing SDS as surfactant [6]. The law of Lamber-Beer is assumed to be applicable and the extinction coefficient is given as 30.3 ± 0.2 mL mg^−1^ cm^−1^ at 1035.3 nm. The π-plasmon resonance energy in the UV-region was measured to calculate the diameter of the tubes [7]. This resonance energy lies at about E = 4.85 eV corresponding to a wavelength of 255 nm. UV-Vis spectroscopy has also been used as a tool to determine the degree of functionalization after oxidative acid treatment indirectly [8]. However, the groups have not been specifically and directly quantified. Only changes of the UV-Vis-spectrum after chemical treatment were recorded to see if the UV-active nanotube sidewall structure was disturbed. Peaks at 800, 660, 580, 510, and 480 nm were attributed to such disturbances. The absorption change of a CNT dispersion after adsorption of nucleotides has also been measured by UV-Vis spectroscopy [9]. A manganese-salen catalyst was covalently bound via a phenylester linkage to acid oxidized CNTs to get a solid state catalyst for the oxidation of cyclohexene with TBHP (tert-butylhydroperoxide) [10], and the amount of grafted manganese-salen complex was quantified by UV-Vis-analysis of a filtrate after the grafting reaction. Using dispersed SWCNTs to form conducting films, the amount of residual surfactant (SDS) slightly changes the spectral pattern of the film in the range between 495 and 2500 nm [11].

The specific analytical problem discussed and solved in the present work is the quantification of adsorbed oxidizing substances like molecular elemental bromine Br_2_ and oxygen O_2_ on brominated multi-walled CNTs (MWCNTs) as a sum parameter and the differentiation between bromine, or halogens in general except iodine, and oxygen or other oxidizing agents than halogens. For comparison, unbrominated pristine MWCNTs were also analyzed for adsorbed oxidants like residual oxygen.

**Figure 1:**
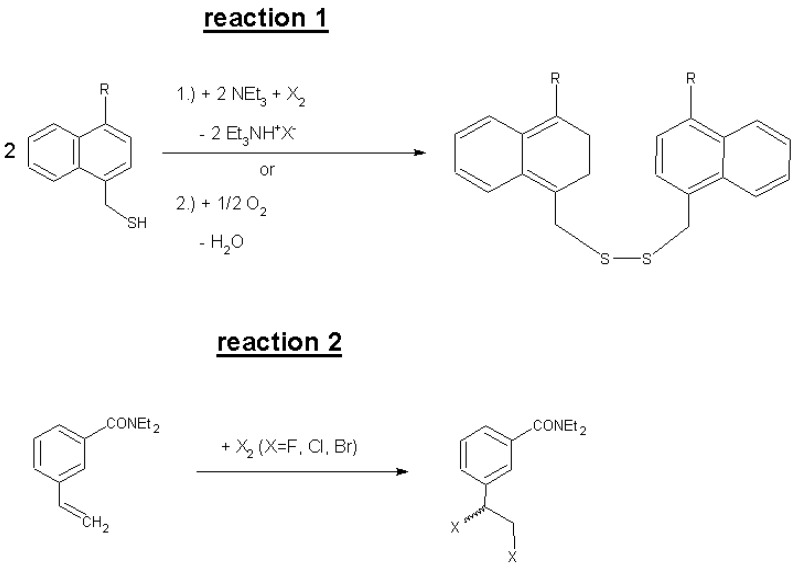
Reactions of substituted (R = organic monovalent rest) 1-naphthylmethylmercaptans with halogens or oxygen and of a substituted 3-styrenecarboxamide with halogens (all at room-temperature (RT)).

For UV-spectrometric quantification and differentiation, specific UV-active reagents were synthesized. Naphthalene derivatives containing the thiol group (substituted 1-naphthylmethylmercaptans) were used for the analysis of all oxidants. Those mercaptans react with all halogens, oxygen, and most other oxidizing groups and molecules forming the corresponding UV-active disulfides (see reaction 1 in Figure 1 with R = H or 2,2,2-trifluoroethoxymethyl, X = F, Cl, Br, or I). Such a thiol reagent with fluorine as marker atom was used for the purpose of subsequent elemental analysis by XPS of the CNTs after derivatization, washing, and drying. This allows the determination of the fraction of the total bromine content of brominated CNTs that is covalently bound in C_sp3_–Br bonds [12]. Such bromine atoms could possibly be substituted by the thiol nucleophile under the reaction conditions, so the thiol is depleted with concomitant release of HBr into the solvent.

A dedicated styrene derivative with a substituted carboxamide group at the benzene ring (3-vinyl-N,N-diethylbenzamide) was used to quantify explicitly free elemental bromine or other halogens except iodine. The product of this reaction will be the racemic 1,2-dibromoethylstyrene derivative, see reaction 2 in Figure 1.

A decrease in the concentration of the reagent in a CNT suspension,* i.e*., the reaction mixture, as well as an increase of the concentration of soluble product are monitored by changes of the UV-extinction of a diluted aliquot of the reaction mixture.

Plotting the functions *E = f*(*t*) for both analyzes and applying an assumed kinetic law, which fits quite well, allows to calculate the content of adsorbed halogens or generally oxidants on the CNTs. The rate constant *k* and the reaction half-life are additionally derived from the *E = f*(*t*) function fitting the only variable parameter *k*. In addition, simple two-variable or three-variable fitting procedures are introduced as examples, because only if linear, quadratic, or cubic polynoms are used, the reaction half-lives can be calculated manually and mathematically formulated simply and analytically in a closed form.

The analytical task of the present work was to develop a method for accurate, sensitive and selective quantification of bromine that is only adsorbed to CNTs, not bound covalently. Such adsorbed or intercalated bromine was expected to be a considerable fraction of the total bromine content resulting from chemical bromination of CNTs as described by Hanelt* et al*. [12].

In addition, adsorbed oxidants and covalently bound oxidizing functional groups like hydroperoxides or oxetanes (cyclic peroxides) on CNTs require progress in chemical analysis. The adsorption of elemental oxygen, ozone, or even atomic oxygen to CNTs has been discussed in the literature, mostly in theoretical papers. Calculations by Khorrampour* et al*. show that elemental oxygen is adsorbed parallel to the CNT surface. Moreover, chemisorption takes place and the density of states at the Fermi level is significantly altered as well as the local geometry [13]. SWCNTs with their interior space filled with oxygen molecules should exhibit CNT-length dependent increasing HOMO-LUMO energy gaps according to theoretical calculations [14]. The formation of ether groups or epoxides, only the latter being susceptible to substitution reactions with nucleophiles like thiols, is only expectable if CNTs are reacted at room temperature with atomic oxygen [15]. NEBM calculations using DFT-LDA showed that the kinetics and likewise the activation energy of oxetane formation after oxygen chemisorption depend on the type of the CNT (armchair, zigzag,* etc*.) [16]. The energy of the exothermic physisorption of O_2_ on SWCNTs was calculated to be about −45 kJ/mol, with the oxygen molecule being at a distance of about 295 pm from the CNT surface. In addition, a charge transfer from the CNT to the O_2_ molecule was predicted [17]. Physisorption of oxygen mostly takes place at structural defect sites, whereas chemisorption occurs preferentially at topological defect sites like Stone-Wales or 7-5-5-7 defects [18]. The physisorption of oxygen on CNTs can be explained by attractive Van der Waals forces and it was shown that SWCNTs have a higher adsorption propensity than planar graphene layers (HOPG), as evidenced by thermal desorption spectroscopy [19]. The amount of adsorbed oxygen is pressure dependent. It was shown by XPS that a low partial pressure of about 1.3 × 10^−9^ bar does not lead to detectable oxygen adsorption [20].

These findings from the literature show that oxidized and/or brominated CNTs or CNTs adsorbed with oxygen and/or bromine species are materials already broadly discussed in theory but only scarcely analyzed quantitatively apart from XPS and thermo desorption analysis. The indirect UV-spectrometric analysis presented in this work will provide a means to quantify and differentiate the analytes adsorbed bromine and oxygen.

## 2. Results and Discussion

### 2.1. Results of the Analyses of Brominated MWCNTs

The results of the analyses are summarized in the Table 1 and Table 2. Table 1 shows the results of the reactions **R4** to **R6**, where the thiols are oxidized to disulfides, whereas Table 2 gives the results of the reactions **R1** to **R3**, where the styrene derivative is used as a selective reagent for bromine.

Furthermore, it is important to point out, that due to the different degree of agglomeration and inhomogenity of each single analyzed CNT sample, the specific values cannot be generalized. Although, each sample was only analyzed once in our study just to give a proof-of-principle, batch-to-batch variations must always be expected.

All reactions **R4** to **R6** are successfully performed, the starting material is consumed and the product forms. The thiols are oxidized and the **disulfides (RSSR, R = organic monovalent rest)** form as the reaction products. However, the thiols are not selective for bromine or halogens, they react with all oxidants, which have a more positive standard redox potential than the disulfide. As consequence, thiols also react with molecular oxygen, peroxides, or hydroperoxides, which may be adsorbed on the CNTs. Even oxidizing functional groups, which are covalently bound to the CNT, like hydroperoxides or cyclic peroxides (1,2-dioxetanes) consume the thiols to form disulfides. In any case, the educt and the product are both dissolved in the reaction solution phase. Thus, the *β* value is not a sole measure of the bromine concentration, but a sum parameter for all oxidizing substances and so for **oxidation equivalents (ox. eq.)**. This is described in Equations (1) and (2):

2*e*^−^ ≅ 2*ox. eq*. ≅ 1*RSSR* ≅ 1*Br_2_* ≅ ½*O_2_* (1)

*β* (*RSSR*) = *β*(*Br_2_*); *β* (*RSSR*) = 2*β*(*O_2_*) (2)

In comparison to* β* as a sum parameter, a second *β* value is necessary, which only takes into account the adsorbed bromine. For this purpose the reagent **13** was synthesized, which as a UV-active aromatic alkene selectively reacts with Br_2_ in an electrophilic addition reaction forming the product **14**. The experiments **R1** to **R3** show, that no reaction between **13** and any of the brominated CNT samples CNT1 to CNT3 takes place, although they have shown oxidizing characteristics.

When reacting with the thiols as reductants the pristine CNTs are expected not to be oxidizing or generally reactive themselves due to possible free radical sites on the CNTs. Free radicals on CNTs can be excluded as the reactive sites because CNTs are known to be radical scavengers themselves [21]. The chemisorption of oxygen on CNTs, which means the formation of oxetanes (cyclic peroxides) as covalently bound oxidizing groups, that also react with the reducing thiols, is enhanced by UV-irradiation [22], so this mechanism can be supposed to play a role in the partial oxidation of CNTs at the open atmosphere by day-light.

**Table 1:** Analytical results of the reactions **R4** to **R6** (oxidation of thiols **16** or **11** to disulfides **17** or **12**) with the calculated reaction parameters.

R No.	Reagent	CNT sample with weight *m_C_* and *V_j_* and *f*	*β* (μmol/g)	*k* (L∙mol^−1^∙h^−1^), single param. + temperature	*τ* (h), *above:* from *k below:* from *a*, *b*, *c*	Iteration parameters a, b, c multiparameter fitting	
**R4**	4-(2,2,2-Trifluoro-ethoxy-methyl)-1-naph-tha-linemethanthiol (**11**) + 1.00 mL tri-ethylamine	0.0127 g **CNT1** *f* = 40, *V_j_* = 0.000250 L	5601 ± 87 ***P* = (R_F_S)_2_** ≅ 1* Br_2_	275 ± 16 ***P*** T = 297 K 138 ± 12 ***A*** T = 297 K	1.328 ± 0.077 ***P***	a (L∙mol^−1^∙h^−3^)	−8.834 ***A***
					1.709 ***A***	b (L∙mol^−1^∙h^−2^)	117.7 ***A***
						c (L∙mol^−1^∙h^−1^)	−72.48 ***A***
**R5**	1-Naphthaline-methan-thiol (**16**) + 1.0 mL tri-ethylamine	0.0589 g **CNT3** *f* = 37, *V_j_* = 0.000270 L	3388 ± 165 ***P* = (RS)_2_** ≅ 1* Br_2_	1535 ± 154 ***P*** T = 295 K	0.232 ± 0.023 ***P***	a (L∙mol^−1^∙h^−3^)	8883 ***P***
					0.250 ***P***	b (L∙mol^−1^∙h^−2^)	−6854 ***P***
						c (L∙mol^−1^∙h^−1^)	2582 ***P***
**R6**	1-Naphthaline-methan-thiol (**16**) + 1.0 mL tri-ethylamine	0.0940 g **CNT0** *f* = 5, *V_j_* = 0.00100 L	207 ± 20 ***P* = (RS)_2_** ≅ 2* O_2_	2041 ± 117 ***P*** T = 296 K	1.531 ± 0.088 ***P***	a (L∙mol^−1^∙h^−3^)	0 ***P***
					1.682 ***P***	b (L∙mol^−1^∙h^−2^)	795.6 ***P***
						c (L∙mol^−1^∙h^−1^)	518.6 ***P***

**Table 2:** Analytical results of the reactions **R1** to **R3** (brominated CNTs with styrene derivative **13**) with the calculated reaction parameters.

R No.	Reagent	CNT sample with weight *m_C_* and *V_j_* and *f*	*β* (μmol/g)	*k* (L∙mol^−1^∙h^−1^) single param. + temperature	*τ* (h)	Iteration parameters a, b, c multiparameter fitting	
**R1**	3-Vinyl-N,N-diethylbenzamide (**13**)	0.1216 g **CNT1** *f* = 200, *V_j_* = 0.00100 L	0	0 T = 295 K	∞	a (L∙mol^−1^∙h^−3^)	0
					∞	b (L∙mol^−1^∙h^−2^)	0
						c (L∙mol^−1^∙h^−1^)	0
**R2**	3-Vinyl-N,N-diethylbenzamide (**13**)	0.1585 g **CNT2** *f* = 200, *V_j_* = 0.00100 L	0	0 T = 295 K	∞	a (L∙mol^−1^∙h^−3^)	0
					∞	b (L∙mol^−1^∙h^−2^)	0
						c (L∙mol^−1^∙h^−1^)	0
**R3**	3-Vinyl-N,N-diethylbenzamide (**13**)	0.1249 g **CNT3** *f* = 10, *V_j_* = 0.00100 L	0	0 T = 296 K	∞	a (L∙mol^−1^∙h^−3^)	0
					∞	b (L∙mol^−1^∙h^−2^)	0
						c (L∙mol^−1^∙h^−1^)	0

The physical effects of adsorption of molecular bromine on CNTs have also been already well discussed in the literature. It is known, for example, that a partial charge transfer from the CNT to the bromine molecule takes place after physisorption forming a partial cationic and hence oxidizing CNT and Br_2_^−^ anions [23,24], the latter paper dealing especially with activated carbon. The results of theoretical calculations show, that after physisorption of Br_2_, which was determined to be a slightly exothermic process with about −10 kJ/mol, the Br–Br bond length increases, the IR stretching frequency decreases and about 0.14 electrons are transferred each bromine molecule [25]. The binding strength of adsorbed bromine is further dependent on the density of states (DOS) at the Fermi level, metallic CNTs with a higher DOS physisorb molecular bromine more strongly than semiconducting CNTs, for example [26].

The result that the aromatic alkene **13** does not react with the brominated MWCNTs is a further hint, that the adsorbed or intercalated bromine is mostly present as reduced and monoanionic Br_2_^−^ molecules, which do not add to the C=C bonds of alkenes like **13** in an electrophilic addition reaction. As already mentioned above, the CNT reduces Br_2_ to form Br_2_^-^, whereas the CNTs are oxidized to CNT^+^ counterions, which are now oxidants themselves. They can now react with reductants like thiols. The oxidizing potential of the brominated CNTs remains, whether the bromine directly or the first partially oxidized CNT finally oxidizes the thiols.

The results in Table 1 also show, that the high temperature brominated CNTs contain more oxidation equivalents than CNTs with bromine, that was only physically adsorbed at room temperature. Even the pristine CNTs exhibit oxidation equivalents on their surface, which amount to about 103 μmol/g, probably as adsorbed oxygen O_2_.

The kinetic data in Table 1 show, that the rate constant k and also the iteration parameters a, b, and c are not material constants or constants specific for one type of reaction. The reason may be that the reaction velocity, especially in the cases discussed here, where redox and addition reactions are performed, which in homogeneous solution phase chemistry are normally very fast, is not determined by the fast chemical reaction itself, but by the slower diffusions in the CNT aggregates. Because of the diffusion kinetics as determining factor on the desorption of bromine and oxygen from the CNT surface in the reaction suspension, the speed of the magnetic stirrer may also influence the reaction velocity, so stirring speed optimizations should be taken into account in the future. Therefore *k*,* a*,* b*, and *c* may be a function of the stirring speed and of the degree of dispersion, aggregation, and bundling of the CNTs. Moreover, the introduced simple kinetic models [see differential equations in formulae (23) and (27)] do not take into account the intrinsic oxidation and addition reaction mechanisms, but can only give the rate constants *k* or *a* to *c* as global constants, which may be functions of several mechanism specific constants of partial reactions.

**Figure 2:**
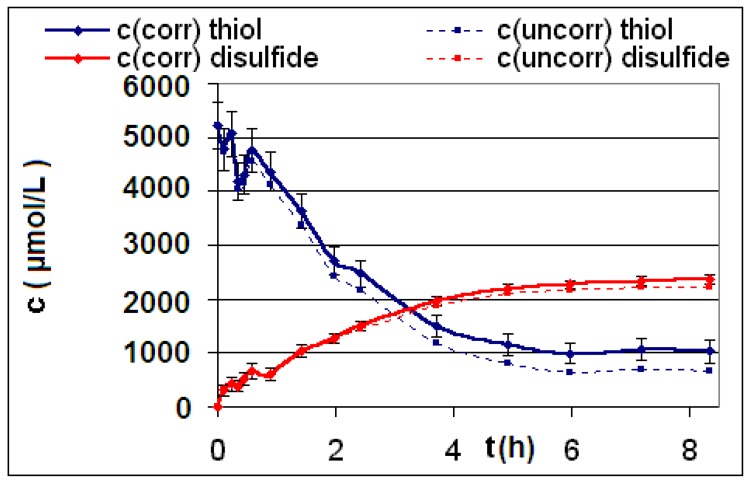
Reaction **R4** comparison *c_corr_*(*t*)*/c_uncorr_*(*t*).

**Figure 3:**
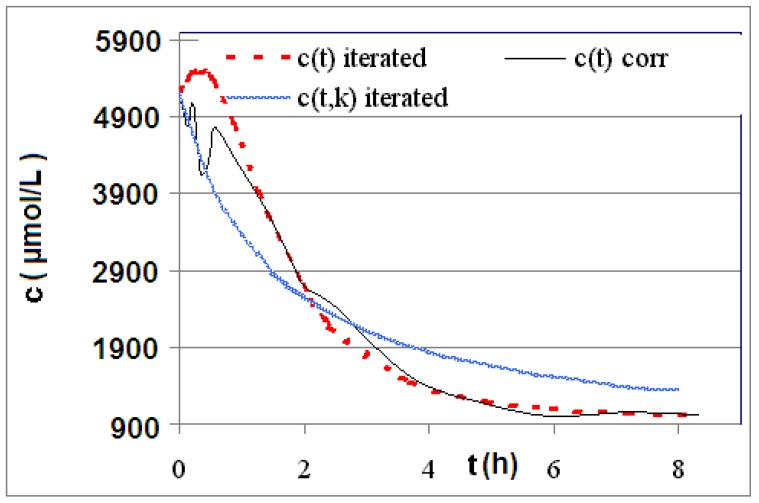
Reaction **R4** comparison *c_corr_*(*t*)*/c_iterated_*(*t*)*/c_iterated_*(*t,k*) only for educt thiol *A*.

**Figure 4:**
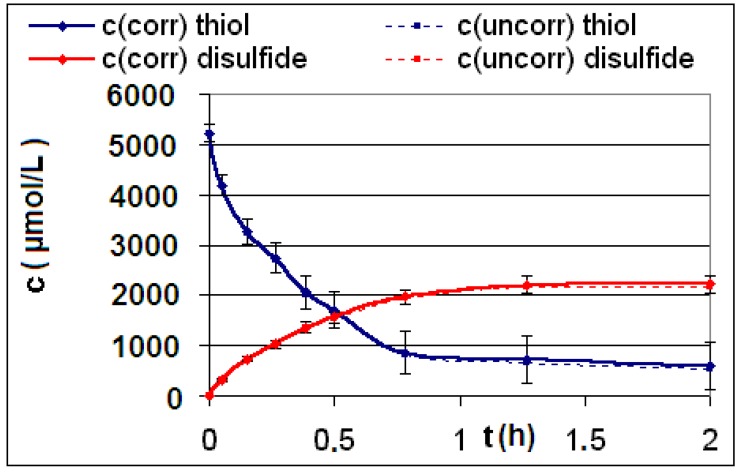
Reaction **R5** comparison *c_corr_*(*t*)*/c_uncorr_*(*t*).

**Figure 5:**
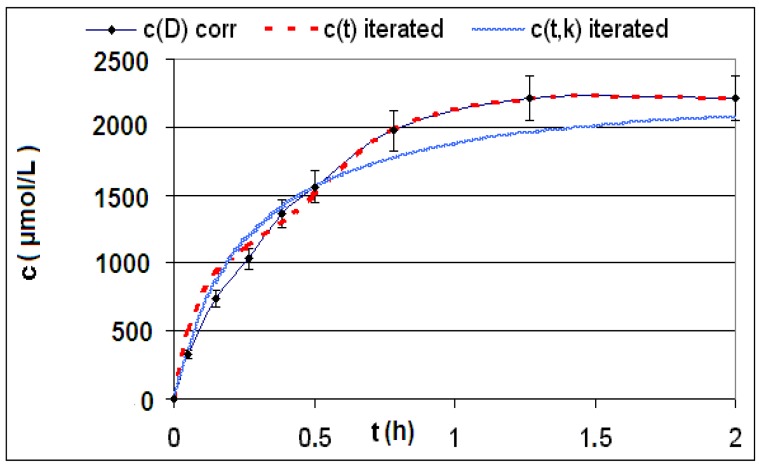
Reaction **R5** comparison *c_corr_*(*t*)*/c_iterated_*(*t*)*/c_iterated_*(*t,k*) only for product *P*.

**Figure 6:**
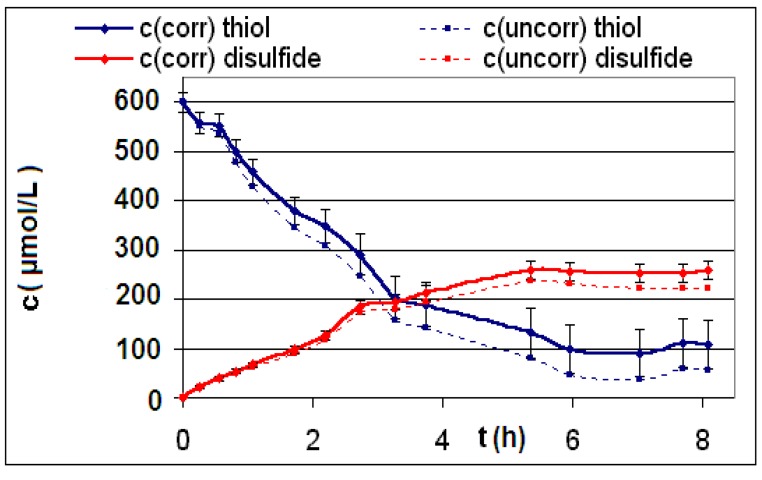
Reaction R6 comparison *c_corr_*(*t*)*/c_uncorr_*(*t*).

**Figure 7:**
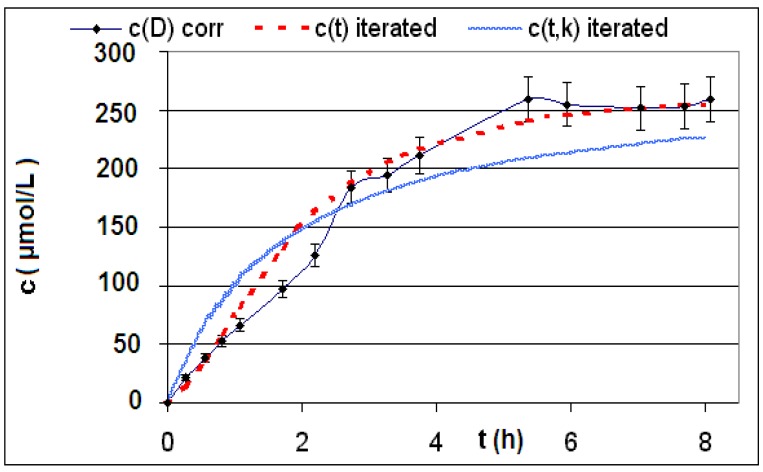
Reaction **R6** comparison *c_corr_*(*t*)*/c_iterated_*(*t*)*/c_iterated_*(*t,k*) only for product *P*.

**Figure 8:**
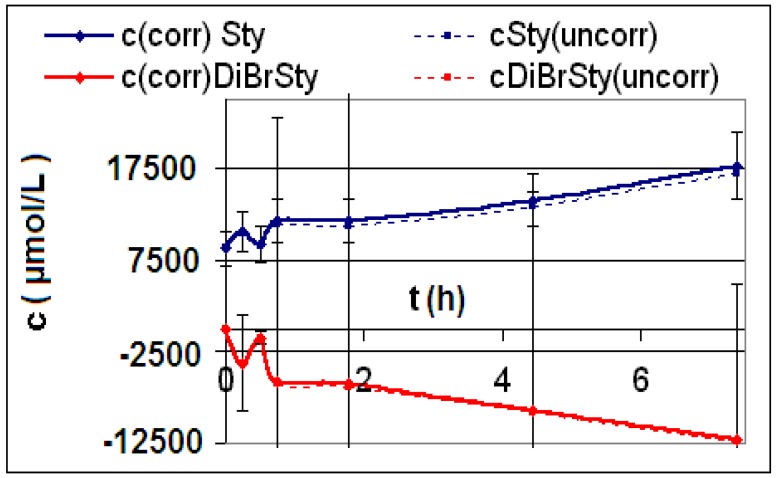
Reaction **R1** comparison *c_corr_*(*t*)*/c_uncorr_*(*t*).

**Figure 9:**
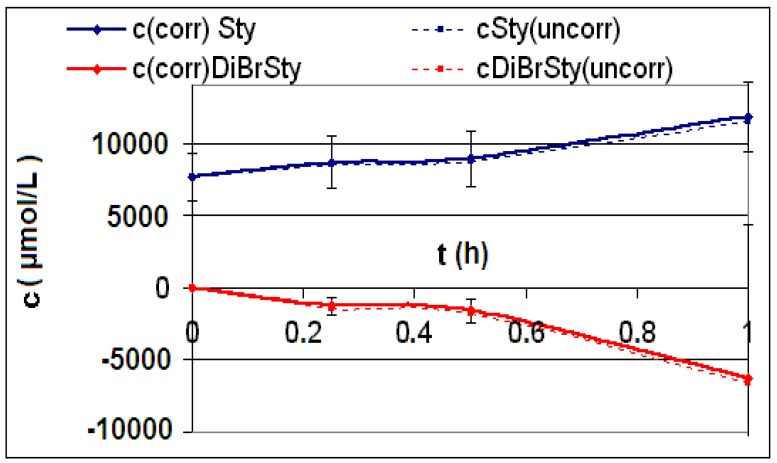
Reaction **R2** comparison *c_corr_*(*t*)*/c_uncorr_*(*t*).

**Figure 10:**
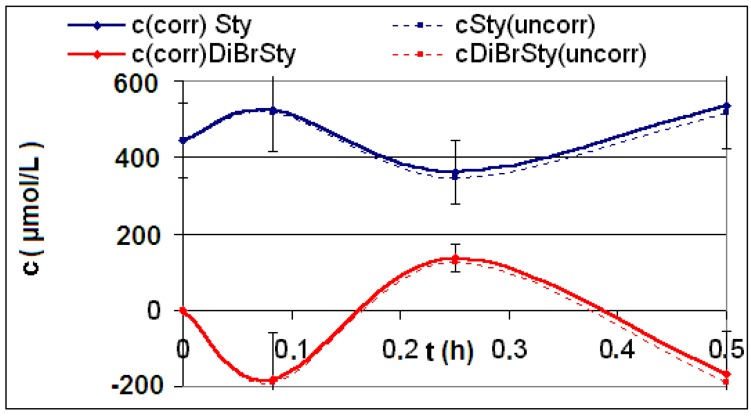
Reaction **R3** comparison *c_corr_*(*t*)*/c_uncorr_*(*t*).

The measured and calculated concentration profiles *c*(*t*) of the reactions **R4** to **R6** are depicted in Figure 2, Figure 3, Figure 4, Figure 5, Figure 6 and Figure 7 including the fitted and iterated curves and the uncorrected *c*(*t*) values for comparison. Table 3 summarizes which figures belong to which reaction. The concentration profiles of the reactions R1 to R3 are depicted in Figure 8, Figure 9 and Figure 10, although no reaction takes place in these cases.

The iterated curves in Figure 3, Figure 5, and Figure 7 show, that first the mechanistic models for the reactions leading to the differential Equations (23) and (27) and to the integrated Equations (24) and (29) are good, though quite simple, and may be superficial theoretical descriptions for the real mechanism. Secondly, a fitting procedure with two or three of the parameters *a*,* b*, and *c* should mostly be preferred to a single parameter iteration using only *k* as the variable.

**Table 3:** Reactions **R4** to **R6** and the appropriate figures for the concentration profiles *c*(*t*).

Reaction	Corrected and uncorrected concentration profiles *c*(*t*)	Corrected concentration profile and iterated curves
**R4**	Figure 2	Figure 3
**R5**	Figure 4	Figure 5
**R6**	Figure 6	Figure 7

The next point to be discussed is the second possible reaction between the thiols and brominated CNTs. Thiols are reductants and also good nucleophiles, which can substitute bromine that is covalently bound and part of C_sp3_–Br, but not C_sp2_–Br bonds. Due to this possible side reaction the fluorine containing UV-active nucleophilic and reducing reagent **11** is synthesized and introduced in this work. If nucleophilic substitution reactions occur, the fluorine containing thiol **11** will be covalently bound as a thioether and consequently after filtration, washing, and drying the solid CNT sample can be analyzed by XPS, so the fluorine content in atom % or μmol/g is a measure for the surface concentration of covalently bound and substitutable bromine. This concentration can also be determined as follows by inspection of the results of the here-described indirect UV-analysis. If no further reaction but the oxidation of the starting thiol occurs, then the concentration ratio of consumed thiol to formed disulfide is 2:1 within statistical error, as can be predicted by the stoichiometry of the reaction. A decision if substitution reactions between the thiol and electrophilic centers on the CNT took place, which can generally also be reactive functional groups other than C_sp3_–Br, like –COBr, or epoxides, or α,β-unsaturated carbonyl groups, must be made on statistical considerations. A substitution as a second reaction besides the redox reaction can significantly be assumed, if the precondition Equation (5) is fulfilled (one-sided t-test) after β (electrophilic centers) has been calculated by Equation (3) including the errors Equation (4) with α as the risk level (e.g., α = 0.05 = 5%) and n as the number of external calibration standards in the determination procedure of the extinction coefficients, because *s_β_* depends on *s_ε_*.

*β*(*el.  centers*) = *β*(*thiol*) − 2 · *β*(*disulfide*) (3)

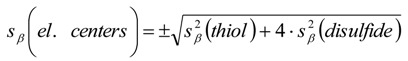
(4)


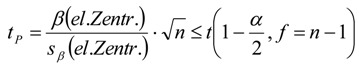
(5)

It turns out that substitution reactions in **R4** to **R6** do not significantly occur, although it was shown, that high temperature brominated CNTs react with a fluorine containing thiol [12]. The difference is that the substitution reaction in reference [12] was carried out in acetonitrile at reflux, whereas R4 to R6 are conducted at room temperature. Consequently, the redox reaction prevails at room temperature in the given time interval between two and nine hours because of the higher reaction velocity.

Finally, the reactions **R4** to **R6** are slower as if conducted in a homogeneous phase like it is described in the synthetic procedures for the substances **12** and **17** (see supplementary section).

This shows that the rates of the heterogeneous reactions **R4** to **R6** appear to be reduced due to the lower diffusivity of the suspended solid phase that requires the dissolved analyte to diffuse to the adsorbing CNT surface. Specific aspects of such heterogeneous diffusion kinetics and the parameters influencing it, although not analyzed on CNTs, are discussed by Shen and Duvnjak [27] for the adsorption of cupric and cadmium cations on corncob particles in solution. For example, the authors point out that faster stirring of the suspension speeds up adsorption kinetics. Adsorption of azo dyes on MWCNTs is discussed by Kuo [28]. The authors derive a pseudo second-order rate constant with a value of (1–6) × 10^−3^ g mg^−1^ min^−1^. The same topic was already studied by Wu in 2007 [29]. The mathematics of diffusion kinetics is thoroughly discussed by Mysels [30]. According to that paper, the geometry of the particles and the surface form and area play a decisive role. The adsorption of lead cations on CNTs, including pseudo second-order rate constants of (4–6) × 10^−3^ g mg^−1^ min^−1^, is discussed by Kabbashi* et al*. [31]. To describe the kinetics of physical adsorption of Cu^2+^ to specific acid activated rubber wood sawdust, Kalavathy* et al*. apply pseudo-first-order or pseudo-second-order models to their data [32], but in contrast to the present paper this is no chemical reaction with an equilibrium that lies almost completely on the side of the products.

In the worst case, the rate constant *k* as calculated in this paper not only differs from reaction to reaction but also from material to material and, maybe, even from batch to batch.

At last, the correctness of the results (the value *β*) of this indirect UV-spectroscopic analysis can only be assured when the *E*(*t*) or rather the *c*(*t*) curves are plotted and sample aliquots are taken and measured as long as the plateau region is not reached. The time scale of the reaction is determined by the reaction velocity itself and the diffusion rate, which can be individual for each material.

### 2.2. Discussion of the Chosen Discontinuous Off-Line Sampling Procedure

The necessity of the described procedure for the indirect UV-analysis of the brominated CNTs has to be pointed out and discussed, because the alternative of a continuous in-line process was not chosen.

The alternative of a continuous in-line process would have been to suspend the CNTs into the reagent solution of known constant volume and let the reaction solution circulate through a flexible tube between the reaction chamber and the cuvette for UV-spectroscopy (with solution inlet and outlet) with the help of a pump, at the end of the flexible tube, where the solution is aspirated, must have been stoppered with a filter adapter. This experimental setup would have prevented the need for the discontinuous removal of the sample aliquots *V_j_* and the correction formulae and mathematics in the following text, which take into account the sample aliquot removal.

But the in-line process as an alternative can have some drawbacks, because the mass of CNTs, the total volume and the amount of UV-active derivatization reagent and so its molar concentration are not independent, because the measured extinction should be in the range of about *E =* 0.07 to *E =* 1.2. Now using this extinction range the extinction coefficient *ε_λ_* determines the concentration of the reagent in the solution. However, in the in-line process the reaction solution is directly measured without dilution or taking aliquots. Those dependencies may lead to unacceptable high reaction volumes, low CNTs masses to be weighed in or low required extinction coefficients. The here-described off-line process has the additional advantage that there is no dead-volume like the flexible tube in a solution circulation setup, the heterogeneous reaction would not take place in the flexible tube.

## 3. Experimental

### 3.1. Materials

For the present work, four differently functionalized types of CNT samples were analyzed. They will be called **CNT0**, **CNT1**, **CNT2**, and **CNT3** in the following.

**CNT0** is the same pristine starting material that was used in referee [12]. It consist of entangled MWCNTs from chemical vapour deposition (CVD) synthesis (Baytubes^®^ C 150P, with carbon purity ≥ 95%, residual metals and metal oxides are Al, Co, Mn) provided by Bayer MaterialScience AG (Leverkusen, Germany).

**CNT1** is a MWCNT sample that was chemically brominated according to the high-temperature bromination procedure by Hanelt* et al*. [12]. This sample contains adsorbed (denoted by Br_2_@CNT) as well as covalently bound bromine (denoted by CNT-Br). CNT1 will therefore also be denoted by Br_2_@CNTBr.

**CNT2** is identical to sample CNT1, but the drying step of the brominated CNT1 in the vacuum oven after washing the CNTs due to the procedure in referee [12] was omitted. The solvents from washing the CNTs were removed only by leaving the sample at the open atmosphere in the hood.

**CNT3 **is a MWCNT sample, on which bromine was only or mostly physically adsorbed and hence it is called Br_2_@CNT. Sample CNT3 was produced as follows: 0.5 g of the starting material CNT0 are weighed into a wide-diameter glass weighing box with a ground glass stopper. A small glass box containing about 6 mL bromine is placed into the glass weighing box besides the CNT powder. The weighing glass box is then stoppered and isolated with PTFE band. The adsorption reaction is a gas phase reaction, and the weighing glass box is opened after four days at room temperature. The small glass box with the liquid bromine is removed and the CNT powder adsorbed with bromine is left at the open atmosphere in the hood for 36 h at RT. Then the material is analyzed by XPS.

The results of the XPS analyses of the four materials are summarized in Table 4.

**Table 4:** Results of X-ray photoelectron spectroscopy (XPS) analyses of the carbon nanotubes (CNT) samples 1–4 prior to indirect UV-analysis.

Material	at % C	at % O	at % Br
CNT0	99.4	0.6	–
CNT1	75.6	6.5	17.9
CNT2	74.1	4.3	21.7
CNT3	97.4	1.0	1.6

An important condition for a successful application of the indirect UV-analytical method presented here, is that no UV-active substances from the material’s synthesis are allowed to desorb during the UV-spectrometrically monitored reaction. The samples therefore have to be washed with appropriate solvents, especially after the chemical bromination procedure described in [12].

### 3.2. Instruments

For UV-absorption measurements, a UV-spectrometer (2101PC, Shimadzu Europa, Duisburg, Germany) and quartz cuvettes of 10.00 mm optical pathlength were used. The UV-range was scanned and measured with a deuterium lamp. The optical resolution was 1 nm.

The XPS-spectrometer was a SAGE 150 (Specs GmbH, Berlin, Germany). XPS measurements were performed with a Phoibos 100 MCD-5 hemispherical analyzer (Specs GmbH, Berlin, Germany) using non-monochromatic Mg K_α_ radiation (11 kV, 20 mA) at a pressure of about 1 × 10^−7^ Pa in the analysis chamber. XPS spectra were acquired in CAE mode. The analyzed surface area was about 3–4 mm^2^.

All NMR measurements (see syntheses of reagents in the supplementary material) were performed at room temperature with an instrument by (Bruker Biospin GmbH, Rheinstetten, Germany). The chemical shifts are given in ppm and the spectra are referenced to the theoretical chemical shifts of the deuterated solvents. ATR-IR spectra were measured with a Nicolet 8700 FT-IR spectrometer (Thermo Scientific GmbH, Germany) using automatic baseline correction and line-smoothing. All uncorrected melting points of the synthesized reagents were determined with a melting point apparatus (Büchi 530 Melting Point, Büchi GmbH, Essen, Germany).

### 3.3. Initial Material Analyses of the CNT Samples

Before starting the UV-spectrometric analysis, the pristine MWCNTs starting material CNT0 and the brominated sample CNT1 were subjected to extended material characterization. The results and micrographs of this analysis have been published in reference [12]. Only the results most relevant for the present work are presented here.

**Figure 11:**
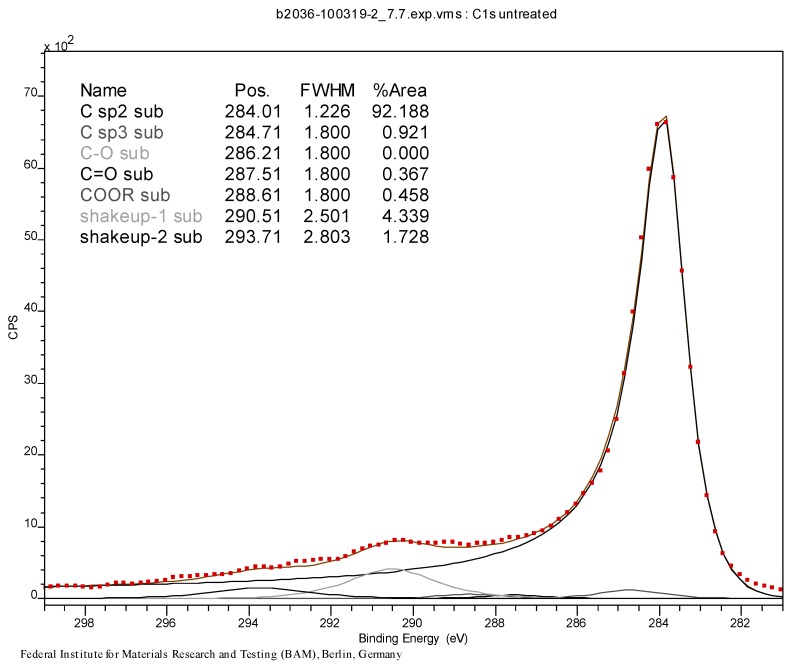
C-1s peak (XPS) of pristine MWCNT sample CNT0.

**Figure 12:**
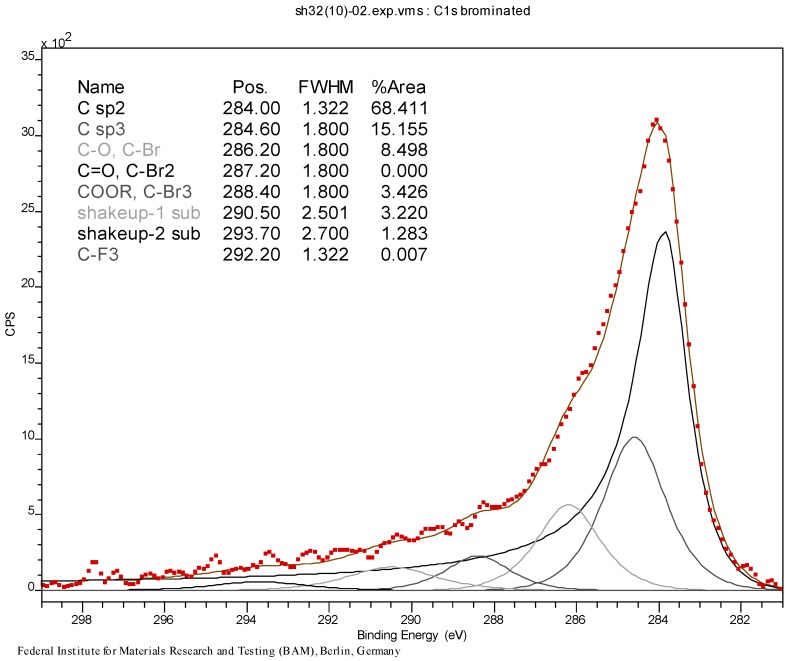
C-1s peak (XPS) of brominated MWCNT sample CNT1.

**Figure 13:**
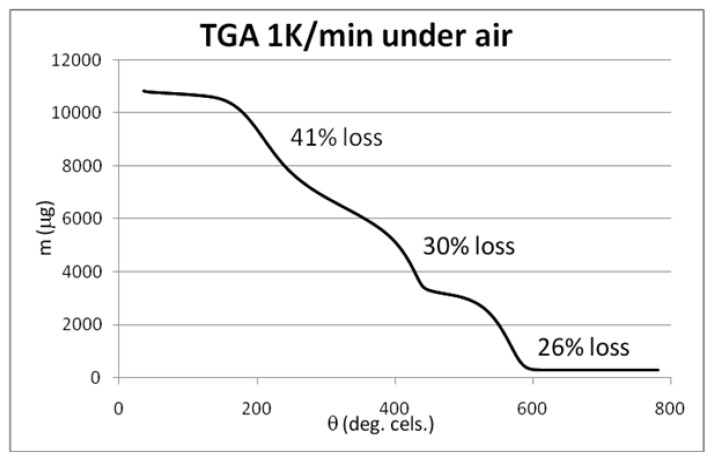
TGA analysis of brominated MWCNT sample CNT1 at 1 K/min.

**Figure 14:**
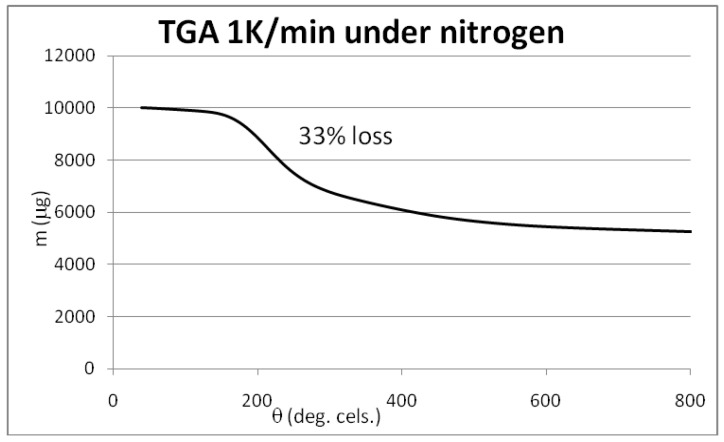
TGA analysis of brominated MWCNT sample CNT1 at 1 K/min under nitrogen.

**Figure 15:**
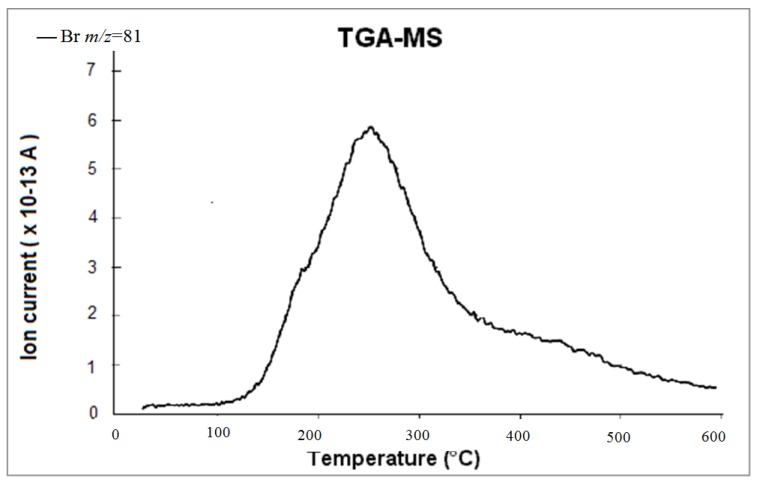
Temperature dependent mass selective (*m/z* = 81) ion current in TGA-MS analysis of sample CNT1.

The results of XPS analysis of the starting and the brominated material CNT0 and CNT1 are given as C-1s spectrum in Figure 11 and Figure 12. The spectra were fitted with respect to chemical shifts of oxidized or brominated carbon. The sp^2^ component of the peak was fitted with an asymmetric Doniac-Sunjic line shape. The thermogravimetric analysis data are shown in Figure 13, Figure 14 and Figure 15. They were acquired at a heating rate of 1 K/min under air and nitrogen as depicted in Figure 13 and Figure 14, respectively. For completion, a TGA-MS selective ion current analysis was performed on CNT1 at 1 K/min under nitrogen with the mass filter set to *m/z* = 81 as depicted in Figure 15.

### 3.4. Syntheses of Reagents and Measurement of UV-Extinction Coefficients

**Figure 16:**
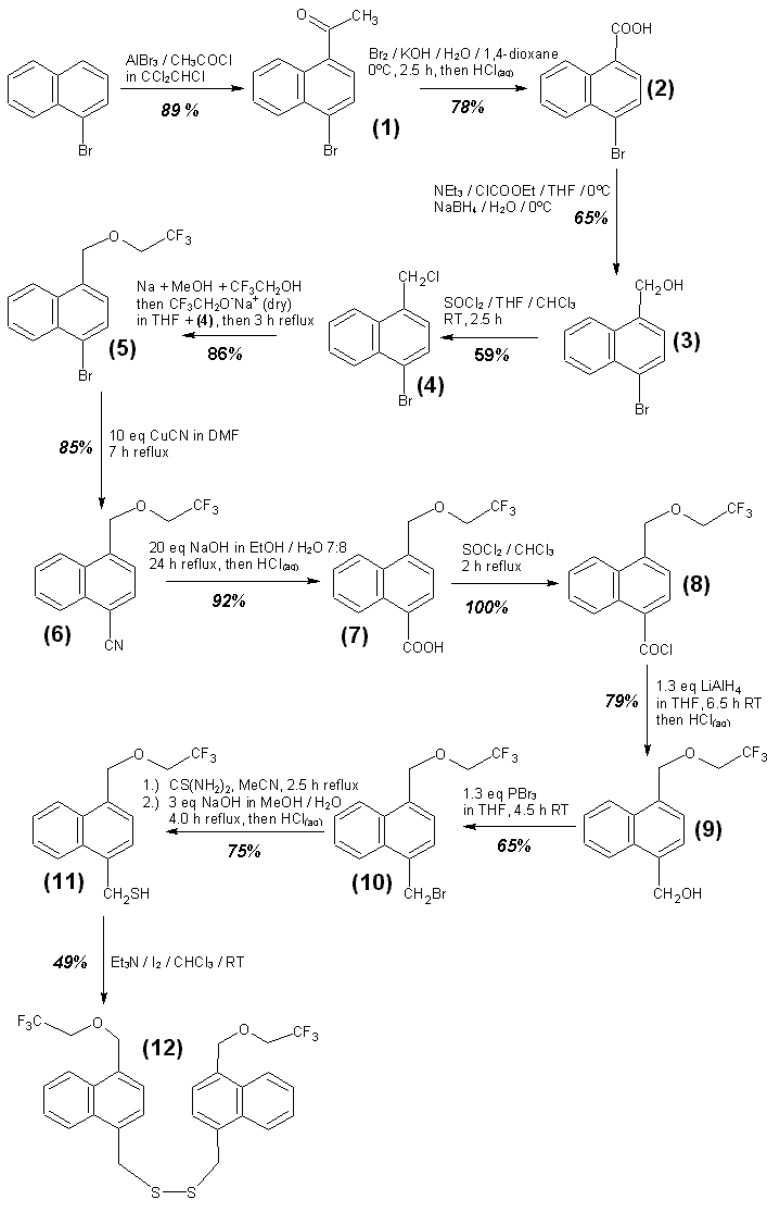
Synthesis steps for substances 1 to 12.

**Figure 17:**
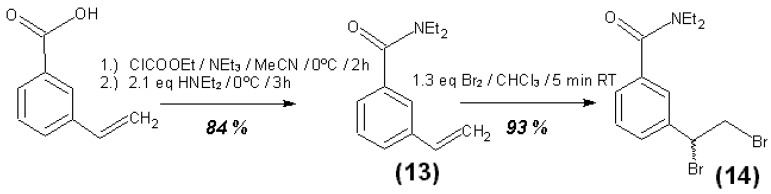
Synthesis steps for substances 13 and 14.

**Figure 18:**
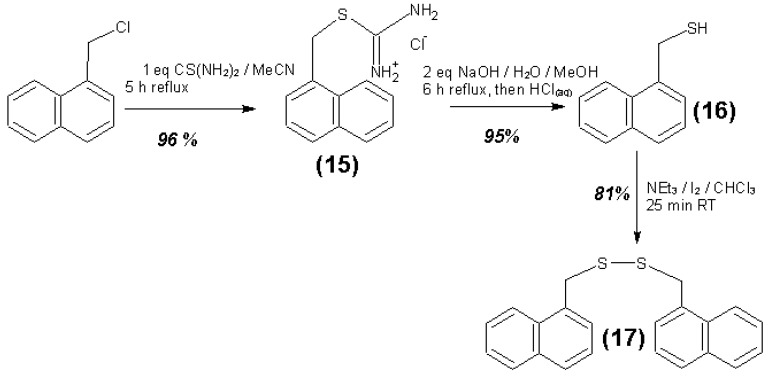
Synthesis steps for substances 15 to 17.

An overview of the synthesis steps for the substances **1** to **17** is given in Figure 16, Figure 17 and Figure 18. Further details of the syntheses are given in the supplementary information.

Table 5 compiles the measured extinction coefficients of the UV-active reagents *ε_λ_* at the wavelengths *λ* of the individual absorption maxima, their statistical concentration detection limits (at 98% confidence level), the absorption maxima of the reagents, and the solvents used.

**Table 5:** Measured extinction coefficients *ε_λ_*, concentration detection limits, absorption maxima *λ* of the UV-active reagents and the solvent used for the reaction and measurement.

Reagent	Solvent	*λ* (nm)	*ε_λ_* (L∙mol^−1^∙cm^−1^)	Concentration detection limit (98% confidence) (μmol/L)
*1-Naphthalenemethan-thiol* (***16***)* + triethylamine*	CH_3_CN	284.5	7672 ± 36	2.13
*1-Naphthalenemethan-thiol* (***16***)* + triethylamine*	CH_3_CN	287.5	6822 ± 11	0.83
*Bis-*(*1-Naphthylmethyl*)*-disulfide* (***17***)* + triethylamine*	CH_3_CN	284.5	15740 ± 157	1.79
*Bis-*(*1-Naphthylmethyl*)*-disulfide* (***17***)* + triethylamine*	CH_3_CN	287.5	16868 ± 161	1.84
*4-*(*2,2,2-Trifluoroethoxymethyl*)*-1-naphthalenemethan-thiol* (***11***)* + triethylamine*	CH_3_CN	289.7	8876 ± 65	2.54
*4-*(*2,2,2-Trifluoroethoxymethyl*)*-1-naphthalenemethan-thiol* (***11***)* + triethylamine*	CH_3_CN	292.7	8196 ± 58	2.41
*Bis-[4-*(*2,2,2-Trifluoroethoxymethyl*)*-1-Naphthylmethyl]-disulfide* (***12***)* + triethylamine*	CH_3_CN	289.7	18974 ± 58	0.45
*Bis-*(*4-*(*2,2,2-Trifluoroethoxymethyl*)*-1-Naphthylmethyl*)*-disulfide* (***12***)* + triethylamine*	CH_3_CN	292.7	19826 ± 55	0.43
*N,N-Diethyl-3-vinylbenzamide* (***13***)	CHCl_3_	241.5	13011 ± 188	2.97
*N,N-Diethyl-3-vinylbenzamide* (***13***)	CHCl_3_	243.5	13681 ± 164	2.46
(*±*)*-N,N-Diethyl-3-*(*1,2-Dibromoethyl*)*-benzamide* (***14***)	CHCl_3_	241.5	6862 ± 61	3.54
(*±*)*-N,N-Diethyl-3-*(*1,2-Dibromoethyl*)*-benzamide* (***14***)	CHCl_3_	243.5	6586 ± 50	3.00

### 3.5. General Procedure

First, the reaction stock solution of the analyte (**11**, **13**, or **16**) was prepared in the required solvent. Its appropriate concentration was calculated according to section 3.6. This requires knowledge of the extinction coefficient of the analyte, see also Table 2. To prepare the reaction solution, in which the UV-spectrometrically traced reaction will be run, an aliquot of the reaction stock solution was removed and pipetted into the reaction vessel, normally a 50 or 100 mL Erlenmeyer flask with a plastic stopper. To this aliquot an additional volume of the pure solvent was pipetted to get the total volume of the reaction solution.

Table 6 shows the specific values of the variables for the six reactions **R1** to **R6** that were performed for the present paper. Each analysis **R1** to **R6** using the different materials CNT0 to CNT3 was only conducted once in this study.

The reagents used and the analytical results of their application to the different CNT samples in the reactions **R1** to **R6** are given in Table 1 **(R4** to **R6**) and Table 2 **(R1** to **R3**). The thiol reagents **11** and **16** were used in the oxidation reactions **R4** to **R6**, whereas the styrene derivative **13** was used as analyte in reactions **R1** to **R3**.

A magnetic stirrer bar was then added to the reaction solution and stirring begun. To get the first extinctions, and hence concentrations, at the time *t* = 0, the first aliquot was removed from the reaction solution, diluted, and measured as described in the following before the CNT sample was added. Subsequently, the CNT sample was weighed into a porcelain weighing boat, poured quickly into the stirred reaction solution (from this moment on the time is counted), and the actual mass *m_C_* of the CNTs in the reaction mixture was calculated by back-weighing the empty porcelain boat. The Erlenmeyer flask was stoppered and aliquots were removed from that moment on in specific regular time intervals. If thiols like **11** or **16** are used as reagents, the reaction must be conducted under argon or nitrogen. In this case, after adding the CNT sample, nitrogen was gently blown into the Erlenmeyer flask over the surface of the solution and the flask was stoppered under nitrogen with a septum with a nitrogen filled balloon on it.

**Table 6:** Variables and values for the preparation of the reaction solutions for the reactions **R1** to **R6**.

Reaction	Reagent	CNT sample	*m_as_* (g)	*V_rs_* (L)	*V_prs_* (L)	*V_s_* (L)	*V_t_* (L)	*m_ar_* (g)
**R1**	*3-Vinyl-N,N-diethyl-benzamide* (***13***)	CNT1	0.6845	0.1000	0.02500	0.05000	0.07500	0.1711
**R2**	*3-Vinyl-N,N-diethyl-benzamide* (***13***)	CNT2	0.6845	0.1000	0.02500	0.05000	0.07500	0.1711
**R3**	*3-Vinyl-N,N-diethyl-benzamide* (***13***)	CNT3	0.1836	0.1000	0.00050	0.06500	0.07000	0.0092
**R4**	*4-*(*2,2,2-Trifluoro-ethoxy-methyl*)*-1-naphthalene-methanthiol* (***11***)	CNT1	0.0572	0.0200	0.01500	0.01500	0.03000	0.0429
**R5**	*1-Naphthalene-methan-thiol* (***16***)* + 1.0 mL tri-ethylamine*	CNT3	0.2583	0.0500	0.01500	0.07500	0.09000	0.0775
**R6**	*1-Naphthalene-methan-thiol* (***16***)* + 1.0 mL tri-ethyl-amine*	CNT0	0.1951	0.1000	0.00500	0.07000	0.07500	0.0098

Notes: *m_as_*, Mass of the analyte in the reaction stock solution in g; *V_rs_*, Volume of the reaction stock solution in liter; *V_prs_*, Volume of the aliquot removed from the reaction stock solution to be diluted in liter; *V_s_*, Volume of pure solvent added to the aliquot *V_prs_* in liter; *V_t_*, Total volume of the reaction solution in liter, *V_t_ = V_s_ + V_prs_*; *m_ar_*, Absolute mass of the analyte in the reaction solution in gram.

The aliquots of the volume *V_j_* were removed from the reaction solution with a full pipette and then diluted by a factor f using volumetric flasks to get the final solution to be measured by UV-spectrometry. All reaction specific variables *f*, *V_j_*, and *m_C_* are given in Table 1 and Table 2 in section 2, together with the analytical results. As two dissolved UV-active analytes are present in the reaction solution simultaneously (starting material and product), their two molar concentrations have to be calculated by measuring two extinctions at two specific wavelengths at each time, which are given in Table 7.

**Table 7:** Wavelengths at which the two extinctions have to be measured at a time in each reaction **R1** to **R6**.

Reaction	*λ_1_* (nm)	*λ_2_* (nm)
**R1**	241.5	243.5
**R2**	241.5	243.5
**R3**	241.5	243.5
**R4**	289.7	292.7
**R5**	284.5	287.5
**R6**	284.5	287.5

When taking the aliquots with the full pipettes one has to pay attention that exactly the volume *V_j_* is taken from the reaction mixture and further diluted, meaning that while emptying the pipette until the meniscus of the liquid reaches the mark the excess of the solution in the pipette must be released back into the reaction solution and not be discarded.

**Figure 19:**
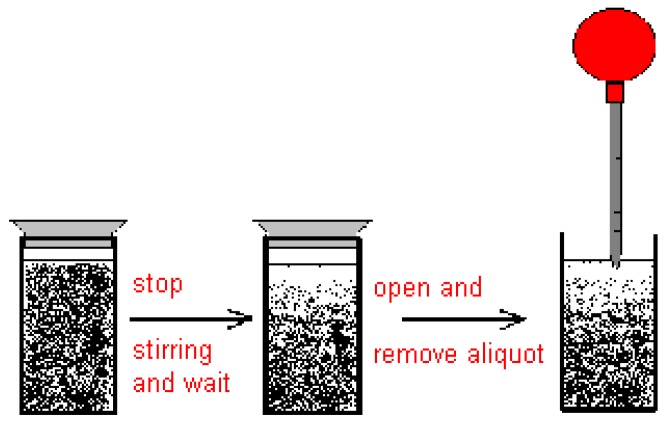
Removal of aliquot from reaction suspension for dilution and UV-measurement.

As the reaction mixture is a suspension and not a solution, the aliquots cannot be taken from the stirred mixture. The sampling was performed 30–45 s after having stopped stirring to give the suspended solids time to settle down for a few millimeters. Then the aliquot was taken with a pipette from the surface of the suspension. This is schematically depicted in Figure 19.

Stirring was restarted after aliquot removal and subsequent aliquot volumes *V_j_* were taken at documented time intervals and diluted as described until the calculated concentrations of the starting material and product (decreasing concentration in the former case, increasing concentration in the latter case) reached a plateau value,* i.e*., successive aliquot concentrations did not change significantly. 

Then the graphs *c = f*(*t*) were plotted and the reaction kinetics was evaluated as shown in Section 3.6, where the mathematical transformations from the two extinctions to the two concentrations at each time are also derived.

### 3.6. Theory and Calculation

#### 3.6.1. Quantification

The values for *m_ar_* and *f* are already specifically given for the reactions run in this study in Table 1 and Table 6, but for a general use *m_ar_* and *f*, the absolute mass of thiol analyte in the reaction solution and the factor by which the aliquot of the volume *V_j_* taken from the reaction solution with the starting total volume *V_t_* has to be diluted, can be calculated according to the Equations (7) and (8), which give optimal values to assure sensible extinctions in the range from *E_max_ − ΔE* to *E_max_* (should be between about 1.2 and 0.5). The stoichiometry of the reaction between a thiol and an elemental halogen like Br_2_ is included in Equations (6) and (7). Furthermore d is the cuvette diameter in cm, *M_ar_* is the molar mass of the UV-active thiol analyte in g/mol, *m_C_* is the weight of the CNT sample in g and SBR is the estimated elemental bromine content (Br_2_) in mol/g, which can also be derived from an XPS analysis with oxygen (*A_O_*) and bromine (*A_Br_*) contents given in atom percentage according to Equation (6), if no further elements but carbon as the main constituent are present. *A_O_* and *A_Br_* are the results in at % oxygen and bromine.

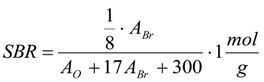
(6)

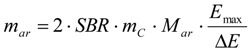
(7)

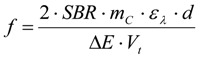
(8)

In the following the surface concentration of bromine *β* in mol/g and the concentrations of the UV-active compounds in the reaction phase at any time *t_i_* are calculated. First, all corrected values are calculated, that take into account the discontinuous removal of aliquots of the volume *V_j_*.

The following general formulae use ***A*** for a starting material, which is consumed like a thiol or styrene derivative in this paper (decreasing concentration of substance **16**, **11**, or **13**), and ***P*** for a product, which forms like a disulfide or dibromoethylbenzene derivative in this paper (increasing concentration of substance **17**, **12**, or **14**).

The two wavelengths *λ*_1_ and *λ*_2_ are the absorption maxima of ***A*** and ***P***, respectively. Thus, *ε_A_*_1_ is the extinction coefficient of ***A*** at *λ*_1_ and *ε_P1_* is the extinction coefficient of ***P*** at *λ*_1_, for example. The value of *s_εA_*_1_ gives the standard deviation (error) of the respective extinction coefficient, for example.

*E*_1__,1_, *E*_1,*i*_, and *E*_1,*n*_ are the first, one intermediate (at the time *t_i_*), and the last extinction measured at *λ*_1_ in this example. Two extinctions *E*_1__,*i*_ and *E*_2,*i*_ have to measured at each time *t_i_*. The last extinctions are measured when the concentrations of both analytes do not significantly change anymore. The variable *n* is the total number of concentration measurements or calculated points in the concentration profile of each one compound.

First, the surface concentration of bromine *β_Br__2_* from the UV-analysis in mol/g is calculated, which later can be compared to the estimated or calculated (see equation 1) value of SBR from a prior XPS analysis.

*β_Br__2_* is determined using the concentration profile of the starting material ***A*** or of the product ***P***. The formula for the calculation of *β_Br__2_* from the concentration profile of the consumed thiol already contains a factor of ½ (2 in the denominator) because of the specific stoichiometry. That factor must be omitted or changed if other reactions are performed like the addition of bromine to the styrene derivative **13**.

Using the concentration profile of the starting thiol ***A***, *β_Br__2_* is according to Equation (9):


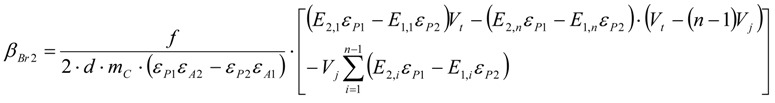
(9)

Using the concentration profile of the product disulfide ***P***, *β_Br__2_* is according to Equation (10):


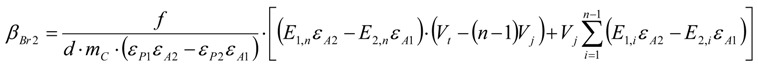
(10)

The factor of ½ is missing here because the stoichiometry between consumed bromine and formed disulfide is 1:1.

According to Equation (11) the concentration of the thiol ***A*** in the reaction mixture at a time *t_k_* (measurement number k) is:


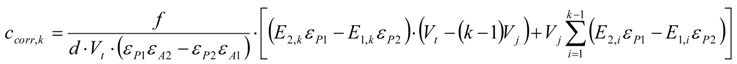
(11)

According to Equation (12) the concentration of the disulfide ***P*** in the reaction mixture at a time *t_k_* (measurement number k) is:

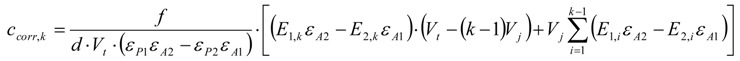
(12)

One special point has to be mentioned only if thiols are used as starting materials. Due to the fact that thiols are prone to aerial oxidation, the synthesized thiol can already be partially oxidized, meaning that it already contains some percent (normally about 5% to 7%) of disulfide. Therefore, it is very important to measure the extinction coefficients of the thiol and disulfide immediately after their synthesis to get the values of the pure reagents. Secondly, only the values of the bromine concentration calculated from ***P*** and the concentrations of disulfide in the reaction mixture have to be corrected by subtraction of the concentration of disulfide, which is already present from the beginning due to the impure thiol reagent. This correction makes it possible even not to use a thiol stored under nitrogen or argon. Equation (13) gives the term *β_rd_* (residule disulfide) to be subtracted from Equation (10) and Equation (14) gives the term *c_rd_* to be subtracted from Equation (12). The concentrations of the thiol do not need to be corrected in this way.

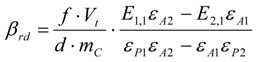
(13)

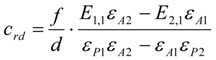
(14)

Due to the presence of four extinction coefficients with their own errors, nine terms (Equations 32–40) are necessary for the calculation of the statistical errors (standard deviations) of the *β* results and concentrations. Terms dependent on* k* have to be calculated individually for each single time *t_k_* (measurement number *k*). The Terms *T*_1_ to *T*_9_ are given in the supplementary section as well as the resulting standard deviations for the surface concentrations β and the corrected concentrations each calculated from the concentration profile of the reacting analyte ***A*** or the formed product ***P***.

The following formulae give the concentrations of ***A*** and ***P*** at a time *t_k_* (measurement number *k*) and bromine surface concentrations *β* without the corrections due to the discontinuous removal of sample aliquots from the reaction solution for comparison. Usually, the corrected values of the concentrations do not equal the uncorrected, this is only true for the first measurement (*k* = 1). Compare Equations (15) with (11), and Equations (16) with (12).

The uncorrected concentration of the starting material ***A*** is according to Equation (15):

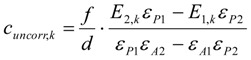
(15)

The uncorrected concentration of the product ***P*** is according to Equation (16):

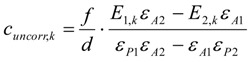
(16)

The uncorrected bromine surface concentration calculated from the concentration profile of the thiol **A** including the stoichiometry-specific factor ½ is according to Equation (17):

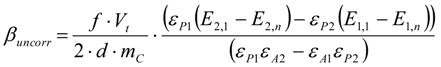
(17)

The uncorrected bromine surface concentration calculated from the concentration profile of the disulfide ***P*** is according to Equation (18):

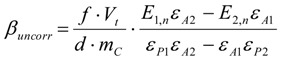
(18)

For the calculation of the statistical errors of the uncorrected variables Equations (15) to (18) the Equations (45) to (48) are given in the supplementary section.

A decision can be made on statistical considerations if the corrections in all the preceding formulae due to the discontinuous aliquot removal must be made or can be omitted, so that the uncorrected terms can be used. Equations (19) and (20) show the general restrictions for the corrections at each *t_k_* with 1 ≤ *k* ≤ *n* in the case of the analyte concentration and the bromine surface concentration, respectively.

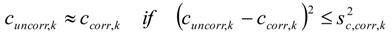
(19)

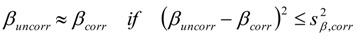
(20)

#### 3.6.2. Reaction Kinetics and Modeled Mechanism

In this chapter simple models for the heterogeneous surface reaction mechanisms are introduced, from which differential equations for the reaction velocities can be derived. Integration yields the theoretical time dependencies of the molar concentrations *c*(*t*) of the consumed starting material ***A*** or the formed product ***P*** in the reaction mixture. A single-parameter fitting procedure results in the rate constant *k*, from which the half-life of the reaction can also simply be calculated. Two and three parameter fittings lead to three constants, from which the reaction half-life can still be calculated by an analytical formula.

First, an additional variable *B_0_* in mol/l must be introduced, because the surface bromine concentration* β* is given in mol/g, which is a different physical unit than mol/L and consequently has to be transformed according to Equation (21). The simplification is that the bromine adsorbed or intercalated is treated as if it was homogeneously dissolved in the liquid phase.

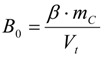
(21)

Reaction kinetics with respect to the formed product *P*

First, the differential equation, its integrated form and the derived formulae for the kinetic variables using the concentration profile of the formed product ***P*** are introduced.

The chemical reaction is the oxidation of a thiol by bromine forming a **disulfide (RSSR)** according to the known and simple chemical Equation (22):

2 *RSH* + *Br_2_* → 2 *HBr* + *RSSR* (22)

With ***A*** as the thiol and ***P*** as the disulfide again, the following differential Equation (23) can be derived [*A*_0_ *= c_thiol,corr_*(*t =* 0 h) *= c_thiol,uncorr_*(*t =* 0 h)] for the concentration increase of the product ***P***:

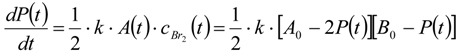
(23)

Integration gives *P*(*t*) in Equation (24):

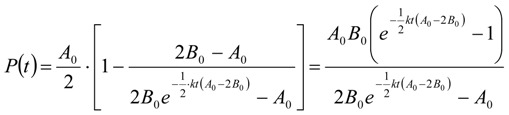
(24)

An excess of the thiol has to be assured, so that *A*_0_* >* 2*B*_0_.

To derive the rate constant *k* in the one-parameter fitting procedure, a term *y_i_ = kt_i_* will be derived from Equation (24) and *k* is given by a simple linear regression *k = dy_i_/dt_i_* according to Equation (25):

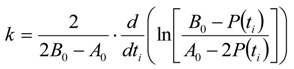
(25)

According to the theory of linear regression, *k* is finally given by (49) and its error by (50), as outlined in the supplementary section.

The reaction half-life *τ* in Equation (26) with *P*(*τ*)* =* ½*B_0_* is:

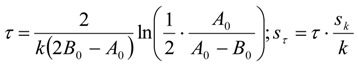
(26)

The two-parameter and three-parameter fitting procedures for a manual calculation are also introduced in the supplementary section. 

Reaction kinetics with respect to the consumed starting material *A*.

The differential equation for the reaction velocity of the consumption of ***A*** is given in Equation (27) and shows no dependence on ***P***(*t*) but only on *A*_0_*, B*_0_, and ***A***(*t*). The velocity of the consumption of the starting material ***A*** is hence proportional to the actual concentration of the starting material ***A***(*t*) and to the right term in Equation (27), which also decreases with time:

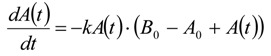
(27)

The right term in brackets is the concentration of the still present reactive sites on the CNTs in mol/L, if the adsorbed or intercalated bromine is taken for a covalently bound reactive site for simplicity.

With the time dependent term *T*_11_(*t*) Equation (27) transforms to the general differential Equation (28):

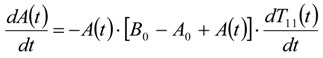
(28)

After integration of Equation (27) ***A***(*t*) is given in Equation (29):

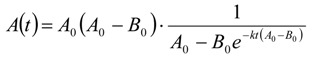
(29)

In comparison to Equation (25) *k* is given by Equation (30) because of the different kinetics:

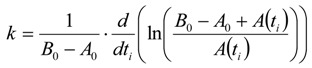
(30)

The half-life *τ* is given by Equation (31):

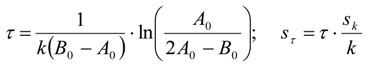
(31)

To calculate the half-life from the iteration parameters *a*, *b*, and *c*, see also the supplementary section. 

## 4. Conclusions

The indirect UV-spectrometry method described here is a viable tool for the specific quantification of adsorbed oxidizing substances on CNTs or solid surfaces in general like oxygen or bromine and their differentiation using UV-active thiols and styrene derivatives, the former react with all oxidants whereas the latter react selectively with molecular bromine. The decrease in concentration of the dissolved analyte in the reaction suspension is monitored by taking aliquots from the supernatant solution, diluting them and measuring the extinction. The course of the concentration per time is then plotted and important parameters besides the surface concentration generally of redox equivalents in µmol/g like the rate constant and the reaction half-life are derived from these data points by a fitting procedure and iteration. The results of the analyses of brominated MWCNTs show, that the measured surface concentrations of adsorbed bromine including adsorbed oxygen lie between about 3300 µmol/g and 5600 µmol/g. Even pristine MWCNTs appear to have adsorbed oxygen in a concentration of about 103 µmol/g. The comparative experiments with the styrene derivatives show, that no reaction occurred with the brominated MWCNTs, which is a further evidence for the already-known fact that molecular bromine oxidizes CNTs forming Br_2_^−^ anions, which do not react with styrene derivatives in an electrophilic addition reaction, whereas the equally formed oxidized and partially cationic CNTs as counterions have now oxidizing character and react with the reducing thiols forming disulfides. The nucleophilic thiols do not react with potentially electrophilic C_sp3_–Br groups in the brominated CNTs under the reaction conditions (room temperature) in the given time interval, in which the redox reaction is completed (2–8 h).

The general method introduced here is applicable to brominated, oxidized or functionalized CNTs as well as to other solid samples, as long as they are insoluble in the reaction solvent and no UV-active impurities are desorbed from the surface into the reaction solvent.

## Supplementary Materials

Supplementary File 1
