# Supplementary material for: UV Spectrometric Indirect Analysis of Brominated MWCNTs with UV Active Thiols and an Alkene—Reaction Kinetics, Quantification and Differentiation of Adsorbed Bromine and Oxygen
Source: Materials (Basel). 2013 Jul 24;6(8):3035–63. doi: 10.3390/ma6083035 (PMC5521233; doi:10.3390/ma6083035)
Supplement: Supplementary File 1 [file materials-06-03035-s001.pdf]

Materials **2013**, *6*, 3035-3063; doi:10.3390/ma6083035

OPEN ACCESS

materials

ISSN 1996-1944

www.mdpi.com/journal/materials

Article

Supporting Information

Syntheses of All Reagents

1. 4-Bromo-1-Acetonaphthone

84.0 g dry aluminum bromide (315 mmol) are placed into a 1 L ground-glass Erlenmeyer flask with a long magnetic stirrer (about 7 cm). 500 mL trichloroethylene are added as solvent afterwards. The reddish solution is cooled to 0 °C. Now a 50 mL dropping funnel with pressure equalizing glass tube on the side is placed on top of the ground-glass flask. The funnel is filled with 21.33 mL acetyl chloride (300 mmol). While stirring fastly the acetyl chloride is dropped into the reaction solution at 0 °C. The funnel is washed with another portion of 10 mL trichloroethylene. The funnel is now filled with 40.00 mL 1-bromonaphthalene (286 mmol). When addition of acetyl chloride is complete, the 1-bromonaphthalene is dropped into the solution at 0 °C during 20 min. The reaction mixture now is a greyish greene suspension. The funnel is washed again with 10 mL trichloroethylene. The mixture is warmed to RT during 30 min and stirred at RT for a further 1.5 h period.

The suspension is poured on a mixture of 500 mL ice, 250 mL water and 200 mL concentrated hydrochloric acid under stirring. The lower organic phase is separated while the aqueous phase is reextracted with 200 mL trichloroethylene. The organic phases are combined, dried over sodium sulphate and filtered. The solvent is removed at the rotary evaporator. The raw product is further purified by elution on a silica gel pad (h = 5 cm, d = 1 0 cm) with petrolether (40–60 °C):ethyl acetate 9:1 (V/V). The main fraction is further purified by adding 2 g of activated carbon and stirring for 5 min. After filtration and solvent removal with the rotary evaporator one gets 63.40 g (89% yield) of a slightly brownish oil of an aromatic odour.

^{1}H-NMR ($CDCl_3$, 600 MHz): δ = 2.61 (s, 3H, CO**CH$_3$**), 7.52–7.57 (m, 3H, 3*C**H**), 7.64 (d, 1H, C**H**, J = 7.8 Hz), 8.20 (dd, 1H, C**H**, J = 8.1 Hz, J = 3.3 Hz), 8.68 (dd, 1H, C**H**, J = 7.5 Hz, J = 2.1 Hz).

^{13}C-NMR ($CDCl_3$, 150 MHz): δ = 29.7 ($COCH_3$), 126.2/127.2/127.5/127.9/128.2/128.36/128.41/130.9/131.9/134.8 (6***C**H + 4***C**), 200.6 (**C**OCH$_3$).

ATR-IR (cm^{-1}): 593,622,650,681,762,793,826,869,951,1015,1104,1137,1180,1200,1240,1274, 1307,1351,1377,1418,1505,1563,1584,1681,2921,3000,3049,3061.

2. 4-Bromo-1-Naphthoic Acid

104 g sodium hydroxide (2.6 mol) are placed into a 1 L ground-glass Erlenmeyer flask with a long magnetic stirrer (about 7 cm) and dissolved in 400 mL water. The solution is cooled to 0–5 °C. Now a 50 mL dropping funnel with pressure equalizing glass tube on the side is placed on top of the ground-glass flask and filled with 39.2 mL bromine (766 mmol). During 30 min all the bromine is dropped into the

stirred sodium hydroxide solution at 0 to 5 °C. Now a 100 mL dropping funnel with pressure equalizing glass tube on the side is placed on top of the ground-glass flask filled with 62.96 g of 4-bromo-1-acetonaphthone **1** (252.7 mmol) dissolved in 30 mL 1,4-dioxane. This solution is dropped into the reaction solution during 15 min at 0 to 5 °C while stirring. The funnel is washed with 10 mL 1,4-dioxane. The ice bath is removed and the mixture stirred for further 2.5 h at RT.

The greyish greene suspension is diluted up to a total volume of 3 L with water. The suspension is then slowly treated dropwise with 224 mL hydrochloric acid *w*(HCl) = 37% under stirring. The mixture turns into a white, thick and clumpy suspension, which is filtrated by suction portionwise. The solid raw product is redissolved in 1.5 L ethyl acetate: diethyl ether 2:1 (V/V). The clear organic solution is extracted with 500 mL water. The organic phase is then separated and dried over Na_2SO_4. After filtration the solvent is removed at the rotary evaporator. The solid is shortly treated in 500 mL boiling methanol. The still hot suspension is filtered by suction. The filtered-off solid is one part of the total product yield. The filtrate is now slowly poured into 750 mL water while stirring at RT. The solution turns into a thick, white suspension, which is again filtered by suction. Both filtered-off solids are combined and dried over P_2O_5 in the desiccator. One gets 49.54 g (78% yield) of a light greyish white powder of a slight aromatic odour.

Melting point: 169 °C–170 °C.

^{1}H-NMR (DMSO-d$_6$, 600 MHz): δ = 7.67 (m, 2H, 2*C**H**), 7.90 (d, 1H, C**H**, *J* = 7.8 Hz), 8.01 (d, 1H, C**H**, *J* = 7.8 Hz), 8.17 (dd, 1H, C**H**, *J* = 7.8 Hz, *J* = 1.2 Hz), 8.94 (dd, 1H, C**H**, *J* = 8.4 Hz, *J* = 1.2 Hz), 13.28 (s, 1H, COO**H**).

^{13}C-NMR (DMSO-d$_6$, 150 MHz): δ = 126.2/126.9/127.0/127.9/128.0/128.3/129.2/130.0/131.3/131.8 (6***C**H + 4***C**), 168.1 (**C**OOH).

ATR-IR (cm^{-1}): 554,598,660,737,762,784,833,902,956,1002,1036,1121,1147,1165,1189,1205, 1251,1279,1311,1356,1375,1406, 1428,1454,1508,1565,1576,1580,1695,2974.

3. 4-Bromo-1-Naphthalenemethanol

48.43 g 4-bromo-1-naphthoic acid **2** (192.9 mmol) are placed into a 1 L ground-glass Erlenmeyer flask with a long magnetic stirrer (about 7 cm) and dissolved in 350 mL dry THF. 26.9 mL triethylamine (193 mmol) are also added. The solution is cooled to 0 °C. Now a 50 mL dropping funnel with pressure equalizing glass tube on the side is placed on top of the ground-glass flask and filled with 18.48 mL ethylchloroformate (193.3 mmol), which is dropped into the solution under stirring at 0 °C during 10 min. Slowly the by-product triethylammoniumchloride separates as white crystals. The thick suspension is stirred at 0 °C for further 3 h. The $HNEt_3^+Cl^-$ is removed by suction filtration and washed with 4*50 mL ice cold dry THF. The filtrate still must be cooled in a 0 °C ice bath. Now the ice cool filtrate is dropped portionwise with a pipette to a stirred solution of 10.95 g sodium borohydride (289.4 mmol) in 500 mL ice water. After some time the CO_2 production begins. When addition of the reactants is complete the suspension is stirred at RT for 30 min. To destroy the excess sodium borohydride 50 mL concentrated hydrochloric acid are now added (evolution of hydrogen). Then 150 mL water and 250 mL diethylether are added, the phases are separated and the aqueous phase is reextracted with another 250 mL diethylether portion. The combined organic phases are washed with 200 mL $NaOH_{(aq)}$ *w*(NaOH) = 10%, dried over Na_2SO_4 and filtered. The solvent is now removed in the vacuum. The oil is treated with 250 mL petrol ether (40–60 °C). Scratching with a

glass stick leads to crystallisation. The petrol ether is decanted and the solid treated with 80 mL boiling ethyl acetate. To this 950 mL n-heptane are added. After rubbing with a glass stick the suspension is put aside for 4 h at −21 °C in the refridgerator. The crystals are filtered by suction and washed with 2*100 mL n-heptane at −21 °C. The filter cake is dried at the open atmosphere over night. The filtrate is evaporated at the open atmosphere in the fume hood until only about 250 mL are left. The second crystal fraction is removed by suction filtration, washed with 2*50 mL n-heptane (RT) and dried at the open air. The two fractions are combined to yield 29.79 g (65% yield) light brown crystal needles.

Melting point: 39–40 °C.

^{1}H-NMR (DMSO-d6, 600 MHz): δ = 4.98 (s, 2H, C**H**$_2$OH), 5.51 (s, 1H, CH$_2$O**H**), 7.50 (d, 1H, J = 7.8 Hz, C**H**^{2}), 7.62 (ddd, 1H ,^3J = 8.4 Hz ,^3J = 7.1 Hz ,^4J = 1.1 Hz, C**H**^{7}), 7.66 (ddd, 1H, ^3J = 8.4 Hz, ^3J = 6.9 Hz, ^4J = 0.9 Hz, C**H**^{6}), 7.84 (d, 1H, J = 7.2 Hz, C**H**^{3}), 8.12 (d, 1H, ^3J = 8.4 Hz, C**H**^{8}), 8.17 (d, 1H, ^3J = 8.4 Hz)

^{13}C-NMR (DMSO-d6, 150 MHz): δ = 60.7 (**C**H$_2$OH), 120.8/124.3/124.8/126.76/126.77/127.3/ 129.5/130.9/131.8/138.3 (6***C**H + 4***C**)

ATR-IR (cm^{-1}): 564,631,749,787,825,901,934,997,1031,1073,1109,1155,1195,1226,1257,1284, 1311,1371,1448,1506, 1567,1594,2858,2914,2968,3049,3074,3285.

4. 4-Bromo-1-Chloromethylnaphthalene

29.37 g (123.9 mmol) 4-bromo-1-naphthalenemethanol **3** are suspended in 200 mL chloroform:THF 1:1 (V/V) in a 500 mL one-necked round-bottomed flask. Under stirring 9.89 mL thionyl chloride (136.3 mmol) are added dropwise at RT. The suspension becomes a homogeneous solution already after only a few drops of $SOCl_2$ have been added. After the complete addition the solution is stirred at RT for further 2.5 h.

The solvents and excess thionyl chloride are removed at the rotary evaporator. The dark oil slowly solidifies while cooling to RT after scratching with a spatula. The dark raw product is recrystallized in 70 mL diisopropylether (cooling at −21 °C). After that the filtered solid is taken up in 100 mL n-heptane and crunched with a spatula. The solid is filtered-off by suction again and washed with 3*50 mL n-heptane of RT. The filtrate is evaporated nearly to dryness and the solid is taken up in 50 mL n-heptane again. This product fraction is again filtered by suction. The combined fractions are dried at the open air to get 18.53 g (59% yield) of light yellowish to white crystal powder.

Melting point: 69−70 °C.

^{1}H-NMR (CDCl$_3$, 600 MHz): δ = 4.97 (d, 2H, J = 7.8 Hz, C**H**$_2$Cl), 7.30−8.36 (m, 6H, 6*C**H**).

^{13}C-NMR (CDCl$_3$, 150 MHz): δ = 43.9 (**C**H$_2$Cl), 123.9−133.3 (6***C**H + 4***C**).

ATR-IR (cm^{-1}): 544,571,609,654,688,716,737,759,781,829,874,898, 920,943,1025,1116,1163,1200, 1234,1256,1325,1381,1423,1451,1510,1519,1567,1577,1589,1622,1659,1815,1842,1866,1910,1934,1962, 2980,3013,3046,3062,3083.

5. 4-Bromo-1-(2,2,2-Trifluoroethoxymethyl)-Naphthalene

First the reagent sodium 2,2,2-trifluoroethanolate is synthesized. For this 6.89 g sodium (300 mmol) are dissolved in 300 mL dry methanol in a 1 L one-necked round-bottomed flask. When the reaction is complete, 26.0 mL 2,2,2-trifluoroethanol (361.8 mmol) are added under stirring. Then the solvent is

evaporated quantitatively using the rotary evaporator. To remove residual methanol completely, which prevents future generation of the methylether byproduct, and to dry the reagent further, the open flask is put into a vacuum oven for 5 h at 115 °C. A longer heat treatment under vacuum may lead to severe decomposition of the alcoholate forming a grey fine powder.

The white dry sodium salt of 2,2,2-trifluoroethanol is now dissolved in 200 mL dry THF. To this a solution of 17.92 g 4-bromo-1-chloromethylnaphthalene **4** (70.1 mmol) in 250 mL acetonitrile: THF 3:2 (V/V) is added in one portion. The reaction mixture is refluxed for 3 h. A white finely crystalline precipitate of sodium chloride soon forms. To work the suspension up 500 mL water are added to the reaction mixture cooled to RT which leads to a formation of two phases. The upper organic phase is separated. The aqueous phase is reextracted with 2*200 mL ethyl acetate. The combined organic phases are dried over sodium sulphate and filtered to remove the solvent on the rotary evaporator. The raw product is eluted on a silica gel pad (h = 5 cm, d = 10 cm) using petrol ether (40–60 °C). The solvent is removed from the product fractions to get 19.29 g (86% yield) of the pure product as a light yellowish liquid of a slightly benzene-like odour.

^{1}H-NMR ($CDCl_3$, 600 MHz): δ = 3.89 (q, 2H, $^3J_{HF}$ = 8.8 Hz, OC**H**$_2$CF$_3$), 5.05 (s, 2H, C**H**$_2$OCH$_2$CF$_3$), 7.31 (d, 1H, J = 7.2 Hz, C**H**^{2}), 7.63 (ddd, 1H, ^3J = 7.6 Hz, ^3J = 7.2 Hz, ^4J = 1.0 Hz, C**H**^{7}), 7.66 (ddd, 1H, ^3J = 7.2 Hz, ^3J = 5.4 Hz, ^4J = 1.8 Hz, C**H**^{6}), 7.76 (d, 1H, J = 7.8 Hz, C**H**^{3}), 8.09 (dd, 1H, ^3J = 7 .8 Hz, ^4J = 1.2 Hz, C**H**^{8}), 8.35 (dd, 1H, ^3J = 7.8 Hz, ^4J = 1.2 Hz, C**H**^{5}).

^{13}C-NMR ($CDCl_3$, 150 MHz): δ = 66.9 (q, $^2J_{CF}$ = 34.3 Hz, **C**H$_2$CF$_3$), 72.0 (**C**H$_2$OCH$_2$CF$_3$), 124.0/124.1 (1*CH + 1***C**), 124.1 (q, $^1J_{CF}$ = 280 Hz, **C**F$_3$), 127.1/127.3/127.4/127.7/129.1/131.9/ 132.0/132.6 (5*CH + 3***C**).

ATR-IR (cm^{-1}): 553,579,619,671,727,758,783,829,913,963,997,1055,1122,1165,1230,1280,1379, 1436,1460,1510,1541, 1568,1596,1610,2873,2928,3052,3075.

6. 4-(2,2,2-Trifluoroethoxymethyl)-1-Naphthonitrile

To a 1 L one-necked round-bottomed flask are added 18.31 g (57.4 mmol) 4-bromo-1-(2,2,2-trifluoroethoxymethyl)-naphthalene **5** and 400 mL DMF as solvent. To this 51.4 g (574 mmol) copper-I-cyanide are further added as a white powder. The suspension is now stirred at about 137 °C for 7 h.

The suspension has now become brownish green in color. To work the mixture up after cooling down to RT 500 mL chloroform are added to precipitate excess CuCN and also formed CuBr. These salts are filtered-off by suction, the filter cake is washed with 2*50 mL chloroform. The filtrate is poured into a 2 L separation funnel together with 500 mL water and 60 mL 1,2-diaminoethane. After shaken vigorously the phases are separated and the upper blue aqueous phase is discarded, but note that the aqueous phase contains complexed copper cations and cyanide.

The organic phase is washed with another portion of 500 mL water and 60 mL 1,2-diaminoethane. After this the organic phase is finally washed with 6*1 L water to remove residual DMF almost quantitatively. The organic phase is dried over Na_2SO_4, filtered and the solvent removed using the rotary evaporator. The raw product is dissolved in a minimum amount of petrolether (40–60 °C):methyl acetate 9:1 (V/V) and purified by elution over a silica gel pad (d = 10 cm , h = 5 cm) using the same eluent petrolether (40–60 °C):methyl acetate 9:1. One gets 12.99 g (85% yield) of a slightly yellowish thick oil of an intense aromatic nitrile-like odour. After some min at RT the oil solidifies but very slowly

because of the low melting point, which is why the product can either be a solid or semi-solid depending on the temperatur.

Melting point: 31–32 °C.

^{1}H-NMR ($CDCl_3$, 600 MHz): δ = 3.97 (q, 2H, $^3J_{HF}$ = 8.8 Hz, OC**H**$_2$CF$_3$), 5.15 (s, 2H, C**H**$_2$OCH$_2$CF$_3$), 7.57 (d, 1H, J = 7.2 Hz, C**H**^{2}), 7.66 (ddd, 1H , ^3J = 8.1 Hz, ^3J = 6.9 Hz , ^4J = 1.2 Hz, CH^7), 7.69 (ddd, 1H, ^3J = 8.4 Hz, ^3J = 7.2 Hz, ^4J = 1.2 Hz, C**H**^{6}), 7.85 (d, 1H, J = 7.8 Hz, C**H**^{3}), 8.07 (d, 1H, ^3J = 8.4 Hz, C**H**^{8}), 8.24 (d, 1H, ^3J = 7.8 Hz, C**H**^{5}).

^{13}C-NMR ($CDCl_3$, 150 MHz): δ = 67.7 (q, $^2J_{CF}$ = 34.5 Hz, **C**H$_2$CF$_3$), 71.7 (**C**H$_2$OCH$_2$CF$_3$), 110.8 (**C**CN), 117.5 (**C**N), 123.9 (q, $^1J_{CF}$ = 279 Hz, **C**F$_3$), 124.0/124.7/125.8/128.0/128.5/130.6/132.0/132.3/ 138.0 (6***C**H + 3***C**).

ATR-IR (cm^{-1}): 550,667,741,761,772,776,842,852,859,966,974,977,1031,1037,1061,1070,1138, 1153,1170,1213,1236,1297,1365,1385,1444,1455,1476,1516,1590,1680,2226,2851,2901,2929,2966,3065.

7. 4-(2,2,2-Trifluoroethoxymethyl)-1-Naphthoic Acid

7.54 g 4-(2,2,2-trifluoroethoxymethyl)-1-naphthonitrile **6** (28.4 mmol) are added to a 250 mL one-necked round-bottomed flask. To this is added a solution of 23 g sodium hydroxide (575 mmol) in 150 mL ethanol:water 7:8 (V/V). First a suspension forms, which is refluxed for 24 h. During this time the starting material dissolves and the mixture becomes a homogeneous solution. After refluxing the still warm solution is slowly poured into a mixture of 54 mL concentrated hydrochloric acid in 1.2 L water under stirring. A white or slightly light yellowish product precipitates. After cooling to RT the product is filtered-off by suction and washed thoroughly with distilled water until the filtrate is pH neutral and free of chloride. The filter cake is removed and dried over P_2O_5 in the desiccator over night during 14 h. Finally the product is further dried in the vacuum oven at 105 °C for 7 h.

One gets 7.47 g (92% yield) of a slightly sand coloured powder. Melting point: 132 °C.

^{1}H-NMR ($CDCl_3$, 600 MHz): δ = 3.94 (q, 2H, CH$_2$OC**H**$_2$CF$_3$, $^3J_{HF}$ = 8.6 Hz), 5.20 (s, 2H, C**H**$_2$OCH$_2$CF$_3$), 7.61 (d, 1H, C**H**, J = 7.2 Hz), 7.64 (ddd, 1H, C**H**, J = 7.8 Hz, J = 7.2 Hz, J = 0.6 Hz), 7.69 (ddd, 1H, C**H**, J = 7.5 Hz, J = 6.9 Hz, J = 0.6 Hz), 8.11 (d, 1H, C**H**, J = 8.4 Hz) , 8.37 (d, 1H, C**H**, J = 7.2 Hz), 9.13 (d, 1H, C**H**, 8.4 Hz), **The COO*H* proton is not visible (traces of acid in the $CDCl_3$?)**.

^{13}C-NMR ($CDCl_3$, 150 MHz): δ = 67.5 (q, CH$_2$O**C**H$_2$CF$_3$, $^2J_{CF}$=34.1 Hz), 72.3 (**C**H$_2$OCH$_2$CF$_3$), 124.0 (q, CH$_2$OCH$_2$**C**F$_3$, $^1J_{CF}$ = 278.6 Hz), 123.8/124.9/126.5/126.7/126.9/128.0/130.9/131.6/131.7/ 138.3 (6***C**H + 4***C**), 172.8 (**C**OOH).

ATR-IR (cm^{-1}): 560,603,650,672,748,782,785,803,827,858,876,966,988,1066,1119,1152,1175, 1204,1260,1269,1273,1286,1300,1334,1361,1386,1414,1438,1464,1519,1595,1618,1677,2534,2626,2 796,2856,2888,2932,3076,3169.

8. 4-(2,2,2-Trifluoroethoxymethyl)-1-Naphthoyl Chloride

To a 250 mL one-necked round-bottomed flask are added 7.25 g (25.5 mmol) of 4-(2,2,2-trifluoroethoxymethyl)-naphthoic acid **7**, which is dissolved in 50 mL chloroform (dried over $MgSO_4$). 3.0 mL thionyl chloride (41.4 mmol) are added under stirring and the solution is refluxed for 2 h. The solvent including excess $SOCl_2$ is removed quantitatively on the rotary evaporator to get a brownish to white oil in quantitative yield. The product slowly solidifies on standing at RT.

Melting point: 37–38 °C.

^{1}H-NMR ($CDCl_3$, 600 MHz): δ = 3.97 (q, 2H, $CH_2OC\mathbf{H_2}CF_3$, $^3J_{HF}$ = 8.8 Hz), 5.20 (s, 2H, $C\mathbf{H_2}OCH_2CF_3$), 7.63–7.73 (m, 3H, 3*C**H**), 8.08 (d, 1H, C**H**, *J* = 8.4 Hz), 8.53 (d, 1H, C**H**, *J* = 7.8 Hz), 8.78 (d, 1H, C**H**, *J* = 9.0 Hz).

^{13}C-NMR ($CDCl_3$, 150 MHz): δ = 67.8 (q, $CH_2O\mathbf{C}H_2CF_3$, $^2J_{CF}$ = 34.2 Hz), 71.9 ($\mathbf{C}H_2OCH_2CF_3$), 123.9 (q, $\mathbf{C}F_3$, $^1J_{CF}$ = 279.8 Hz), 123.8/124.3/125.7/127.5/129.3/130.3/130.6/131.2/134.4/140.4 (6***C**H + 4***C**), 167.4 (**C**OCl).

ATR-IR (cm^{-1}): 535,560,586,654,739,774,806,837,967,990,1030,1054,1161,1215,1238,1279,1368, 1411,1434,1473, 1519, 1580,1583,1592,1621,1645,1758,2850,2930,3085,3181,3370.

9. 4-(2,2,2-Trifluoroethoxymethyl)-1-Naphthalenemethanol

To a 250 mL one-necked round-bottomed flask are added 3.92 g (13 mmol) 4-(2,2,2-trifluoroethoxymethyl)-1-naphthoyl chloride **8** and dissolved in 60 mL dry THF under nitrogen. At RT 4.3 mL c = 2 mol/L lithiumaluminumhydride in THF (8.5 mmol) are added by a syringe while stirring during 5 min under nitrogen. The reaction solution is stirred under nitrogen at RT for further 6.5 h. To quench the reaction a solution of 2.2 mL concentrated hydrochloric acid in 10 mL water are slowly added under water bath cooling. After this 30 mL water are added. Now the product is extracted with 75 mL diethylether. After separation of the organic phase the organic phase is washed with 2*50 mL water and 1*50 mL sodium carbonate solution $w(Na_2CO_3)$ = 10%. The organic phase is dried over sodium sulphate, filtered and the solvent removed at the rotary evaporator. The raw product is an oil which soon crystallizes during cooling to RT. The solid is resuspended in 25 mL diethylether and the chunks are crunched with a spatula. Now 200 mL n-hexane are added and the suspension is cooled to: −21 °C over night. Then the solid is filtered by suction and washed with 3*20 mL n-hexane of −21 °C. The product is dried at the open atmosphere to get 2.78 g (79% yield) of a light sand coloured crystalline powder.

Melting point : 87–88 °C.

^{1}H-NMR (DMSO-d$_6$, 600 MHz): δ = 4.15 (q, 2H, $CH_2OC\mathbf{H_2}CF_3$, $^3J_{HF}$ = 9.4 Hz), 4.99 (d, 2H, $C\mathbf{H_2}OH$, *J* = 5.4 Hz), 5.10 (s, 2H, $C\mathbf{H_2}OCH_2CF_3$), 5.36 (t, 1H, $CH_2O\mathbf{H}$, *J* = 5.4 Hz), 7.52–7.62 (m, 4H, 4*C**H**), 8.11–8.13 (m, 2H, 2*C**H**).

^{13}C-NMR (DMSO-d$_6$, 150 MHz): δ = 61.1 ($\mathbf{C}H_2OH$), 66.3 (q, $CH_2O\mathbf{C}H_2CF_3$, $^2J_{CF}$ = 32.9 Hz), 71.7 ($\mathbf{C}H_2OCH_2CF_3$), 124.5 (q, $CH_2OCH_2\mathbf{C}F_3$, $^1J_{CF}$ = 279.2 Hz), 123.3/124.2/124.3/125.8/125.9/ 126.6/130.9/131.2/131.5/138.8 (6***C**H+4***C**).

ATR-IR (cm^{-1}): 557,643,666,688,709,751,780,836,964,997,1068,1117,1156,1271,1280,1373,1413, 1440,1467,1517,1646,2849,2884,2903,2929,3081,3183,3367.

10. 4-(2,2,2-Trifluoroethoxymethyl)-1-Bromomethylnaphthalene

A portion of 2.65 g (9.81 mmol) 4-(2,2,2-trifluoroethoxymethyl)-1-naphthalenemethanol **9** is added to a 50 mL Erlenmeyer flask and dissolved in 25 mL dry THF. Then 0.40 mL phosphorus tribromide (4.3 mmol) are added slowly while stirring at RT. The flask is stoppered and the mixture stirred at RT for a further 4.5 h period. Then 50 mL ethyl acetate are added and the organic phase is washed in the separatory funnel with 1*100 mL water and 1*100 mL saturated sodium hydrogencarbonate solution.

The separated and combined aqueous phases are reextracted with 1*75 mL ethyl acetate. The combined organic phases are dried over Na_2SO_4, filtered and the solvent is removed by rotary evaporation. The light brown oil is treated with 50 mL n-hexane and scratched with a spatula. A light brown precipitate forms, which is filtered by suction and washed with 2*20 mL n-hexane. The filtrate is reduced at RT to a volume of about 15–20 mL. The second crystalline fraction is filtered by suction and combined with the first product fraction. The product is dried at 45 °C to 50 °C in the vacuum oven for 2 h to get 2.13 g (65% yield) of a light brownish to grey white fine crystalline powder.

Melting point: 75–76 °C.

^{1}H-NMR ($CDCl_3$, 600 MHz): δ = 3.88 (q, 2H, $CH_2OC\mathbf{H}_2CF_3$, $^3J_{HF}$ = 8.8 Hz), 4.95 (s, 2H, $C\mathbf{H}_2Br$), 5.11 (s, 2H, $C\mathbf{H}_2OCH_2CF_3$), 7.43 (d, 1H, C**H**, J = 7.2 Hz), 7.52 (d, 1H, C**H**, J = 7.2 Hz), 7.62 (ddd, 1H, C**H**, J = 8.1 Hz, J = 7.5 Hz, J = 0.6 Hz), 7.67 (ddd, 1H, C**H**, J = 7.8 Hz, J = 7.2 Hz, J = 0.6 Hz), 8.15 (d, 1H, C**H**, J = 8.4 Hz), 8.21 (d, 1H, C**H**, J = 8.4 Hz).

^{13}C-NMR ($CDCl_3$, 150 MHz): δ = 31.3 ($\mathbf{C}H_2Br$), 67.0 (q, $\mathbf{C}H_2CF_3$, $^2J_{CF}$ = 34.3 Hz), 72.4 ($\mathbf{C}H_2OCH_2CF_3$), 124.1 (q, $CH_2\mathbf{C}F_3$, $^1J_{CF}$ = 279.4 Hz), 124.4/124.5/126.4/126.7/126.8/127.0/131.3/ 132.0/133.6/134.5 (6*$\mathbf{C}$H + 4*$\mathbf{C}$).

ATR-IR (cm^{-1}): 535,606,664,712,735,754,780,804,835,868,985,1018,1045,1132,1154,1179,1201, 1250,1273,1313,1337,1361,1392,1401,1412,1443,1451,1455,1463,1518,1592,1618,2868,2898,2939, 2974,3047,3075.

11. 4-(2,2,2-Trifluoroethoxymethyl)-1-Naphthalenemethanthiol

In a 250 mL one-necked round-bottomed flask 1.08 g (3.25 mmol) 4-(2,2,2-trifluoroethoxymethyl)-1-bromomethylnaphthalene **10** are dissolved in 50 mL acetonitrile. To this solution is added a warm solution of 247.6 mg (3.25 mmol) thiourea in 20 mL acetonitrile. The reaction mixture is now refluxed for 2.5 h.

Then the solvent is removed quantitatively at the rotary evaporator. The solid is redissolved in 50 mL methanol. After that a solution of 0.78 g NaOH (19.5 mmol) in 15 mL water is added. The reaction mixture is refluxed for 4.0 h.

Now most of the methanol is removed by rotary evaporation so that mostly water remains. The basic emulsion is treated with 2.2 mL concentrated hydrochloric acid and the product is extracted in the separatory funnel with 3*50 mL chloroform. The combined organic phases are dried over sodium sulphate, filtered and the solvent is removed by rotary evaporation. The raw product is purified by elution over a silica gel pad (d = 10 cm, h = 5 cm) with chloroform as eluent. The product fraction is collected and the solvent removed quantitatively to get 705 mg (76% yield) of a nearly colourless oil. This oil only slowly solidifies on standing at RT during about two days forming an off-white to white and slightly waxy solid.

Melting point: 42–43 °C.

^{1}H-NMR ($CDCl_3$, 400 MHz): δ = 1.91 (t, 1H, $CH_2S\mathbf{H}$, J = 7.0 Hz) , 3.87 (q, 2H, $CH_2OC\mathbf{H}_2CF_3$, $^3J_{HF}$ = 8.8 Hz), 4.20 (d, 2H, $C\mathbf{H}_2SH$, J = 7.2 Hz), 5.11 (s, 2H, $C\mathbf{H}_2OCH_2CF_3$), 7.43–8.18 (m, 6H, 6*C**H**).

^{13}C-NMR ($CDCl_3$, 100 MHz): δ = 26.6 ($\mathbf{C}H_2SH$), 66.9 (q, $CH_2O\mathbf{C}H_2CF_3$, $^2J_{CF}$ = 34 Hz), 72.5 ($\mathbf{C}H_2OCH_2CF_3$), 124.1 (q, $CH_2OCH_2\mathbf{C}F_3$, $^1J_{CF}$ = 278 Hz), 124.2/124.7/125.3/126.4/126.5/126.8/131.0/ 131.8/132.2/138.2 (6*$\mathbf{C}$H + 4*$\mathbf{C}$).

ATR-IR (cm^{-1}): 550,669,758,797,843,965,994,1056,1116,1164,1279,1367,1390,1436,1516,1592, 1689,2873,2929,3015,3044,3075.

12. Bis-(4-(2,2,2-TTrifluoroethoxymethyl)-1-Naphthylmethyl)-Disulfide

In a 200 mL Erlenmeyer flask 358.3 mg (1.25 mmol) 4-(2,2,2-trifluoroethoxymethyl)-1-naphthalenemethanthiol **11** are dissolved in 30 mL chloroform (dried over $MgSO_4$), to which 0.30 mL triethylamine (2.15 mmol) are added. While stirring at RT a solution of 182.6 mg iodine (0.72 mmol) in 20 mL dry chloroform are added dropwise. When addition is complete the solution is of an orange colour because of the excess iodine. The solution is now poured into a separation funnel and washed with 1*50 mL sodium thiosulphate solution w($Na_2S_2O_3$) = 10% and 1*50 mL water. The organic phase is separated and dried over sodium sulphate, filtered and the solvent is removed by rotary evaporation. The oily raw product is treated with 20 mL methanol. Scratching with a spatula leads to a solidification of the oily raw product to a white solid. The first fraction is filtered-off by suction and the filtrate is reduced to about 10 mL at RT. After further scratching with a spatula and after 2 h at −20 °C a second solid fraction crystallized, which is removed by suction filtration and washed with 1*5 mL methanol of −20 °C. The combined solid fractions are dried over P_2O_5 to get 174 mg (49% yield) of a greyish to white finely crystalline solid.

Melting point: 48–49 °C.

^{1}H-NMR ($CDCl_3$, 400 MHz): δ = 3.85 (q, 4H, 2*$CH_2OC\mathbf{H}_2CF_3$, $^3J_{HF}$ = 8.8 Hz), 3.99 (s, 4H, $C\mathbf{H}_2SSC\mathbf{H}_2$), 5.10 (s, 4H, 2*$C\mathbf{H}_2OCH_2CF_3$), 7.08–8.14 (m, 12H, 12*C**H**).

^{13}C-NMR ($CDCl_3$, 100 MHz): δ = 41.3 ($\mathbf{C}H_2SS\mathbf{C}H_2$), 66.9 (q, 2*$CH_2O\mathbf{C}H_2CF_3$, $^2J_{CF}$ = 34 Hz), 72.5 (2*$\mathbf{C}H_2OCH_2CF_3$), 124.1 (q, 2* $CH_2OCH_2\mathbf{C}F_3$, $^1J_{CF}$ = 278 Hz), 124.5/124.7/126.2/126.3/126.5/ 127.4/131.5/132.0/132.2/133.9 (12***C**H + 8***C**).

ATR-IR (cm^{-1}): 546,571,669,719,756,779,840,877,965,1001,1060,1131,1169,1278,1366,1393, 1439,1462,1477,1516,1595,2846,2901,2928,2950,3014,3045,3062,3080.

13. N,N-Diethyl-3-Vinylbenzamide

In a 250 mL one-necked round-bottomed flask 4.758 g (32.1 mmol) 3-vinylbenzoic acid is dissolved in 70 mL acetonitrile. To this are added 4.48 mL triethylamine (32.1 mmol). The solution is cooled to 0 °C while stirring. At 0 °C 3.06 mL ethylchloroformate (32.1 mmol) are added via syringe while stirring. A white precipitate of triethylammoniumchloride forms. The suspension is further stirred at 0 °C for 2 h, then at 0 °C 6.98 mL diethylamine (67.4 mmol) are added. The mixture is then stirred at 0 °C for 3 h. To work up the suspension is reduced to a volume of about 30 mL at the rotary evaporator. Now 50 mL water, 50 mL saturated sodium chloride solution and 75 mL ethyl acetate are added. The mixture is poured into a separation funnel, shaken and the aqueous phase is discarded. The organic phase is washed with 1*50 mL hydrochloric acid *w*(HCl) = 10%, 2*50 mL water and 1*75 mL saturated sodium hydrogencarbonate solution. The organic phase is then dried over Na_2SO_4, filtered and the solvent quantitatively removed by rotary evaporation to get 5.479 g (84% yield) of a slightly yellowish oil of an intense menthone-like odour, which is pure enough for the purposes in this paper.

^{1}H-NMR ($CDCl_3$, 600 MHz): δ = 0.99 (t, 3H, N[$CH_2C\mathbf{H}_3$][CH_2CH_3], J = 7.2 Hz), 1.13 (t, 3H, N[CH_2CH_3][$CH_2C\mathbf{H}_3$], J = 7.2 Hz), 3.13 (s, 2H, N[$C\mathbf{H}_2CH_3$][CH_2CH_3]), 3.42 (s, 2H,

$N[CH_2CH_3][C\mathbf{H}_2CH_3]$), 5.15 (d, 1H, $CH{=}C\mathbf{H_{cis}}H_{trans}$, J = 10.8 Hz), 5.65 (d, 1H, $CH{=}CH_{cis}\mathbf{H_{trans}}$, J = 17.4 Hz), 6.57 (dd, 1H, $C\mathbf{H}{=}CH_2$, $^3J_{HH\ trans}$ = 17.4 Hz , $^3J_{HH\ cis}$ = 10.8 Hz), 7.11–7.30 (m, 4H, $C\mathbf{H}^{2,4,5,6}$).

^{13}C-NMR ($CDCl_3$, 150 MHz): δ = 12.5 ($N[CH_2\mathbf{C}H_3][CH_2CH_3]$), 13.8 ($N[CH_2CH_3][CH_2\mathbf{C}H_3]$), 38.9 ($N[\mathbf{C}H_2CH_3][CH_2CH_3]$), 42.9 ($N[CH_2CH_3][\mathbf{C}H_2CH_3]$), 114.3 ($CH{=}\mathbf{C}H_2$), 123.7/125.1/126.5/128.2/135.8/137.1/137.4 ($\mathbf{C}H{=}CH_2$ + $\mathbf{C}CH{=}CH_2$ + $\mathbf{C}CO\ N[CH_2CH_3][CH_2CH_3]$ + $\mathbf{C}H^{2,4,5,6}$), 170.7 ($\mathbf{C}O\ N[CH_2CH_3][CH_2CH_3]$).

ATR-IR (cm^{-1}): 564,631,713,753,776,805,910,993,1023,1093,1173,1219,1276,1314,1364,1381, 1429,1460,1462,1579,1633,1696,2876,2934,2975,3055,3088.

14. (±)-N,N-Diethyl-3-(1,2-Dibromoethyl)-Benzamide

In a 250 mL one-necked round-bottomed flask 3.04 g (14.96 mmol) N,N-diethyl-3-vinylbenzamide **13** are dissolved in 50 mL chloroform (dried over $MgSO_4$). While stirring at RT, 1.00 mL bromine (19.5 mmol) are added dropwise during 5 min, after which the mixture is stirred for further 10 min at RT.

The reaction mixture is poured into a separation funnel and washed with 100 mL sodium thiosulphate solution w($Na_2S_2O_3$) = 5%. Then 50 mL saturated sodium chloride solution are also added to lead to a better phase separation. The organic phase is separated and the aqueous phase reextracted with 75 mL chloroform. The combined organic phases are dried over Na_2SO_4, filtered and the solvent is removed quantitatively by rotary evaporation. One gets 5.04 g (93% yield) of the racemic product as a thick oil, which partially solidifies very slowly after standing at RT for days or even weeks, so the melting point can not be determined correctly and seems to be below about 28–30 °C.

^{1}H-NMR ($CDCl_3$, 600 MHz): δ = 0.98 (t, 3H, $N[CH_2C\mathbf{H}_3][CH_2CH_3]$, J = 7.2 Hz), 1.12 (t, 3H, $N[CH_2CH_3][CH_2C\mathbf{H}_3]$, J = 7.2 Hz), 3.11 (s, 2H, $N[C\mathbf{H}_2CH_3][CH_2CH_3]$), 3.42 (s, 2H, $N[CH_2CH_3][C\mathbf{H}_2CH_3]$), 3.87 (dd, 1H, $CHBr\text{-}CBrH_{cis}\mathbf{H_{trans}}$, $^2J_{HH}$ = 10.5 Hz, $^3J_{HH}$ = 10.5 Hz), 3.94 (dd, 1H, $CHBr\text{-}CBr\mathbf{H_{cis}}H_{trans}$, $^2J_{HH}$ = 10.2 Hz, $^3J_{HH}$ = 5.4 Hz), 5.03 (dd, 1H, $C\mathbf{H}Br\text{-}CBrH_{cis}H_{trans}$, $^3J_{HH\ trans}$ = 10.8 Hz, $^3J_{HH\ cis}$ = 5.4 Hz), 7.22–7.32 (m, 4H, $C\mathbf{H}^{2,4,5,6}$).

^{13}C-NMR ($CDCl_3$, 150 MHz): δ = 12.6 ($N[CH_2\mathbf{C}H_3][CH_2CH_3]$), 13.9 ($N[CH_2CH_3][CH_2\mathbf{C}H_3]$), 34.6 ($CHBr\mathbf{C}H_2Br$), 39.1 ($N[\mathbf{C}H_2CH_3][CH_2CH_3]$), 43.0 ($N[CH_2CH_3][\mathbf{C}H_2CH_3]$), 49.7 ($\mathbf{C}HBrCH_2Br$), 125.3/126.7/128.2/128.7 ($\mathbf{C}H^{2,4,5,6}$), 137.2 ($\mathbf{C}CHBrCH_2Br$), 138.5 ($\mathbf{C}CO\ N[CH_2CH_3][CH_2CH_3]$), 170.1 ($\mathbf{C}O\ N[CH_2CH_3][CH_2CH_3]$).

ATR-IR (cm^{-1}): 574,614,630,705,752,803,843,880,930,973,1022,1097,1132,1172,1217,1285,1313, 1364,1367,1381,1428,1469,1630,1692,2875,2934,2975,3028,3055.

15. S-(1-Naphthylmethyl)-Isothiuroniumchloride

40.88 g (231 mmol) 1-chloromethylnaphthalene (melting in a water bath is necessary prior to weighing in) are weighed into a 500 mL one-necked round-bottomed flask. 300 mL acetonitrile are added as solvent followed by 17.62 g (231 mmol) thiourea as a solid. The mixture is refluxed for five hours. While refluxing a white precipitate already forms. The suspension is then cooled to RT and filtered by suction. The filter cake is then washed with 3*50 mL acetonitrile of room temperature. The white solid is dried at the open atmosphere over night to get 56.12 g (96% yield) of a white and finely crystalline powder. Melting point : 226–227 °C (Decomposition).

^{1}H-NMR (Pyridine-d_5, 400 MHz): δ = 5.41 (s, 4H, $CH_2SCN\mathbf{H_2}[N\mathbf{H_2}^+]$), 5.43 (s, 2H, $C\mathbf{H_2}SCNH_2[NH_2^+]$), 7.35–8.31 (m, 7H, 7*C**H**$_{Naphthalene}$).

^{13}C-NMR (Pyridine-d_5, 100 MHz): δ = 34.5 ($\mathbf{C}H_2SCNH_2[NH_2^+]$), 124.3/126.2/126.8/127.4/129.2/ 129.5/129.9/130.2/132.0/134.6 (7***C**H$_{Naphthalene}$ + 2***C**$_{Naphthalene}$ + 1***C**CH_2), 171.5 ($CH_2S\mathbf{C}NH_2[NH_2^+]$).

ATR-IR (cm^{-1}): 678,772,1065,1219,1245,1395,1416,1440,1510,1528,1645,2702,2727,3078, 3171, 3215.

16. 1-Naphthalenemethanthiol

55.18 g (218 mmol) S-(1-Naphthylmethyl)-isothiuroniumchloride **15** is weighed into a 500 mL one-necked round-bottomed flask and 250 mL methanol are added as solvent. To the white suspension a solution of 34.92 g (873 mmol) sodium hydroxide in 70 mL water is poured while stirring. The mixture is refluxed for six hours. During this period the precipitate mostly redissolves. Now the reaction mixture is filtered by suction, the filter cake is washed with 5*15 mL methanol. The filtrate is reduced to a total volume of about 200 mL at the rotary evaporator. To this filtrate 58 mL of concentrated hydrochloric acid (*w*(HCl) = 37%) are slowly poured while stirring. After this a portion of 100 mL water and 50 mL saturated sodium chloride solution are added. The product is extracted in the separation funnel with 3*70 mL dichloromethane. The combined organic phases are washed with 250 mL saturated sodium hydrogencarbonate solution, dried over sodium sulphate, filtered and the dichloromethane is removed quantitatively by rotary evaporation. The product is pure enough for the purposes. One gets 36.13 g (95% yield) of a clear, colourless oil of a penetrant stench. The product has to be stored under nitrogen or argon.

^{1}H-NMR (CD_3CN, 400 MHz): δ = 2.14 (t, 1H, S**H**, J = 7.4 Hz), 4.17 (d, 2H, C**H$_2$**SH, J = 7.2 Hz), 7.39–8.11 (m, 7H, 7*C**H**$_{Naphthalin}$).

^{13}C-NMR (CD_3CN, 100 MHz): δ = 26.8 (**C**H_2SH), 124.8/126.7/126.9/127.1/127.2/128.9/129.8/ 131.7/135.0/138.4 (7***C**H$_{Naphthalin}$ + 2***C**$_{Naphthalin}$ + 1***C**CH_2).

ATR-IR (m^{-1}): 542,579,629,685,736,778,797,862,970,1017,1080,1166,1242,1351,1396,1435,1510, 1596,1689,1809,1929,2566,2856,2938,3010,3047.

17. Bis-(1-Naphthylmethyl)-Disulfide

1.67 g (9.6 mmol) 1-naphthalenemethanthiol **16** are weighed into a 250 mL Erlenmeyer flask together with 1.5 mL triethylamine (10.5 mmol) and dissolved in 40 mL chloroform (dried over $MgSO_4$). Under stirring at RT a solution of 1.28 g iodine (5.03 mmol) in 60 mL dry chloroform is slowly dropped into the reaction solution. Due to the excess iodine the solution turns from colourless to orange at the end of the reaction. The mixture is transferred into a separation funnel with 50 mL chloroform. The organic phase is washed with 1*100 mL *w* = 10% sodium thiosulphate solution, 1*100 mL *w* = 10% hydrochloric acid and 1*200 mL *w* = 10% sodium carbonate solution and finally with 1*100 mL water. The washed organic phase is dried over sodium sulphate, filtered and the solvent is quantitatively removed by rotary evaporation. The slightly yellowish oil is cooled to RT and then scratched with a spatula which leads to crystallization. The raw product is suspended in 50 mL methanol and boiled for 15 min. The suspension is then cooled to −5 °C. The product is removed by suction filtration and washed with 4*15 mL methanol of −5 °C. The product is dried at the open

atmosphere over night to get 1.34 g (81% yield) of a white, microcrystalline powder. Melting point: 105–106 °C.

^{1}H-NMR ($CDCl_3$, 400 MHz): δ = 4.05 (s, 4H, C**H**$_2$SSC**H**$_2$), 7.21–8.09 (m, 14H, 14*C**H**)

^{13}C-NMR ($CDCl_3$, 100 MHz): δ = 41.2 (**C**H$_2$SS**C**H$_2$), 124.0/125.1/125.7/126.1/128.1/128.4/128.7/131.2/132.6/133.8 (14***C**H + 6***C**).

ATR-IR (cm^{-1}): 536,583,741,779,802,864,906,955,974,1014,1138,1166,1225,1266,1353,1394,1442,1510,1595,1813,1940,2932,2962,3010,3043,3084.

Mathematical Section with Additional and Supplementary Formulae Terms T_1 to T_9:

$$T_1 = E_{2,k}\left(V_t - (k-1)V_j\right) + V_j \sum_{i=1}^{k-1} E_{2,i} \quad (32)$$

$$T_2 = E_{1,k}\left(V_t - (k-1)V_j\right) + V_j \sum_{i=1}^{k-1} E_{1,i} \quad (33)$$

$$T_3 = \left(E_{2,k}\varepsilon_{P1} - E_{1,k}\varepsilon_{P2}\right)\cdot\left(V_t - (k-1)V_j\right) + V_j \sum_{i=1}^{k-1}\left(E_{2,i}\varepsilon_{P1} - E_{1,i}\varepsilon_{P2}\right) \quad (34)$$

$$T_4 = \frac{1}{\varepsilon_{P1}\varepsilon_{A2} - \varepsilon_{P2}\varepsilon_{A1}} \quad (35)$$

$$T_5 = \left(E_{1,k}\varepsilon_{A2} - E_{2,k}\varepsilon_{A1}\right)\cdot\left(V_t - (k-1)V_j\right) + V_j \sum_{i=1}^{k-1}\left(E_{1,i}\varepsilon_{A2} - E_{2,i}\varepsilon_{A1}\right) \quad (36)$$

$$T_6 = E_{2,1}\varepsilon_{P1} - E_{1,1}\varepsilon_{P2} \quad (37)$$

$$T_7 = \sqrt{s_{\varepsilon_{P1}}^2\left(T_1 - T_3T_4\varepsilon_{A2}\right)^2 + s_{\varepsilon_{P2}}^2\left(T_2 - T_3T_4\varepsilon_{A1}\right)^2 + T_3^2T_4^2\left(s_{\varepsilon_{A1}}^2\varepsilon_{P2}^2 + s_{\varepsilon_{A2}}^2\varepsilon_{P1}^2\right)} \quad (38)$$

$$T_8 = \sqrt{s_{\varepsilon_{P1}}^2\left(T_4T_6\varepsilon_{A2} - E_{2,1}\right)^2 + s_{\varepsilon_{P2}}^2\left(T_4T_6\varepsilon_{A1} - E_{1,1}\right)^2 + T_6^2T_4^2\left(s_{\varepsilon_{A1}}^2\varepsilon_{P2}^2 + s_{\varepsilon_{A2}}^2\varepsilon_{P1}^2\right)} \quad (39)$$

$$T_9 = E_{1,1}\varepsilon_{A2} - E_{2,1}\varepsilon_{A1} \quad (40)$$

Standard Deviations Calculated from T_1 to T_9

The standard deviation for the surface concentration of bromine calculated from the concentration profile of the thiol **A** including the special factor ½ for this reaction only is according to Equation (41):

$$s_\beta(k=n) = \pm\frac{f\cdot T_4}{2\cdot d\cdot m_C}\sqrt{T_7^2 + V_G^2T_8^2} \quad (41)$$

The standard deviation for the surface concentration of bromine calculated from the concentration profile of the disulfide **P** is according to Equation (42):

$$s_\beta(k=n) = \pm\frac{\beta}{T_5}\cdot\sqrt{s_{\varepsilon_{A1}}^2\left(T_1 - T_4T_5\varepsilon_{P2}\right)^2 + s_{\varepsilon_{A2}}^2\left(T_2 - T_4T_5\varepsilon_{P1}\right)^2 + T_4^2T_5^2\left(s_{\varepsilon_{P1}}^2\varepsilon_{A2}^2 + s_{\varepsilon_{P2}}^2\varepsilon_{A1}^2\right)} \quad (42)$$

The standard deviation of the concentration of the thiol **A** is according to Equation (43):

$$s_{c,corr,k} = \pm \frac{c_{corr,k}}{T_3} \cdot \sqrt{s_{\varepsilon_{P1}}^2 \left(T_1 - T_3 T_4 \varepsilon_{A2}\right)^2 + s_{\varepsilon_{P2}}^2 \left(T_2 - T_3 T_4 \varepsilon_{A1}\right)^2 + T_3^2 T_4^2 \left(s_{\varepsilon_{A1}}^2 \varepsilon_{P2}^2 + s_{\varepsilon_{A2}}^2 \varepsilon_{P1}^2\right)} \quad (43)$$

The standard deviation of the concentration of the disulfide **P** is according to Equation (44):

$$s_{c,corr,k} = \pm \frac{c_{corr,k}}{T_3} \cdot \sqrt{s_{\varepsilon_{A1}}^2 \left(T_1 - T_3 T_4 \varepsilon_{P2}\right)^2 + s_{\varepsilon_{A2}}^2 \left(T_2 - T_4 T_5 \varepsilon_{P1}\right)^2 + T_4^2 T_5^2 \left(s_{\varepsilon_{P1}}^2 \varepsilon_{A2}^2 + s_{\varepsilon_{P2}}^2 \varepsilon_{A1}^2\right)} \quad (44)$$

Errors of the Uncorrected Surface Concentrations and Analyte Concentrations

The statistical error of the uncorrected concentration of the starting material **A** is according to Equation (45):

$$s_{c,uncorr,k} = \pm \frac{c_{uncorr,k}}{T_6} \cdot \sqrt{s_{\varepsilon_{P1}}^2 \left(E_{2,k} - T_4 T_6 \varepsilon_{A2}\right)^2 + s_{\varepsilon_{P2}}^2 \left(\varepsilon_{A1} T_4 T_6 - E_{1,k}\right)^2 + T_4^2 T_6^2 \left(s_{\varepsilon_{A2}}^2 \varepsilon_{P1}^2 + s_{\varepsilon_{A1}}^2 \varepsilon_{P2}^2\right)} \quad (45)$$

The statistical error of the uncorrected concentration of the product **P** is according to Equation (46):

$$s_{c,uncorr,k} = \pm \frac{c_{uncorr,k}}{T_9} \cdot \sqrt{s_{\varepsilon_{A2}}^2 \left(E_{1,k} - T_4 T_9 \varepsilon_{P1}\right)^2 + s_{\varepsilon_{A1}}^2 \left(\varepsilon_{P2} T_4 T_9 - E_{2,k}\right)^2 + T_9^2 T_6^2 \left(s_{\varepsilon_{P1}}^2 \varepsilon_{A2}^2 + s_{\varepsilon_{P2}}^2 \varepsilon_{A1}^2\right)} \quad (46)$$

The statistical error of the uncorrected bromine surface concentration calculated from the concentration profile of the thiol **A** including the stoichiometry specific factor ½ is according to Equation (47):

$$s_{\beta,uncorr} = \pm \frac{V_t}{2 \cdot m_C} \cdot \sqrt{s_{c,uncorr,1}^2 + s_{c,uncorr,n}^2} \quad (47)$$

The statistical error of the uncorrected bromine surface concentration calculated from the concentration profile of the disulfide **P** is according to Equation (48):

$$s_{\beta,uncorr} = \pm \frac{V_t}{m_C} \cdot s_{c,uncorr,n} \quad (48)$$

Formulae for the Kinetics

$$k = \frac{\sum_{i=1}^{n} y_i t_i}{\sum_{i=1}^{n} t_i^2} \quad (49)$$

The statistical error of k is given by Equation (50) with n as number of measured points:

$$s_k = \pm \sqrt{\frac{n}{n-1} \cdot \frac{\sum_{i=1}^{n} \left(y_i - k \cdot t_i\right)^2}{n\left(\sum_{i=1}^{n} t_i^2\right) - \left(\sum_{i=1}^{n} t_i\right)^2}} \quad (50)$$

This linear regression may pose some problems, because sometimes, especially for larger t_i values (longer reaction times, concentration does not change much any more), there are some outliers and a

linear dependency can not be assumed any more. In this case only the first sub*J*ectively chosen values $y_i(t_i)$ can be used for the linear regression. In an alternative procedure, the $P(t)$ function (25) can be directly fitted with k as only parameter to the measured concentrations $c_{corr,k}$.

The **two- and three-parameter fitting procedures for the concentration profile of the product P** are introduced here for manual calculations, for this the exponential term in Equation (24) is written as given in Equation (51) with the new terms $T_{10}(t)$ and $T_{11}(t)$:

$$e^{-\frac{1}{2}kt(A_0-2B_0)} = e^{\frac{1}{2}kt(2B_0-A_0)} = e^{T_{10}(t)} \quad with \quad T_{10}(t) = \frac{1}{2}(2B_0 - A_0)\cdot T_{11}(t) \tag{51}$$

In the case of only one fitted parameter (k) as shown above, the following relationship Equation (52) is valid:

$$T_{11}(t_i) = k \cdot t_i = y_i \quad and \quad \frac{dT_{11}(t)}{dt} = k \tag{52}$$

Using the general term $T_{11}(t)$ the differential Equation (23) can be rewritten as given in Equation (53):

$$\frac{dP(t)}{dt} = \frac{1}{2}\cdot(P(t) - B_0)\cdot(2P(t) - A_0)\cdot\frac{dT_{11}(t)}{dt} \tag{53}$$

The Term $T_{11}(t)$ is now generally written as a cubic term Equation (54) with three variables a, b and c:

$$T_{11}(t) = a\cdot t^3 + b\cdot t^2 + c\cdot t \tag{54}$$

During the fitting process, for example simply using the Microsoft Excel functions, a, b and c are varied until at the optimum $P(t) \approx P(T_{11}(t))$ is valid. It varies from case to case if a three-parameter iteration (a, b and c calculated) or a two-parameter iteration (a = 0.l mol^{-1} h^{-3}) gives the better regression with a correlation coefficient closer to one.

For the calculation of the reaction half-life τ, $T_{11}(t)$ must be calculated with $t = \tau$ as given in Equation (55) to yield the new term $T_{12}(A_0, B_0)$:

$$T_{11}(t=\tau) = \frac{2}{2B_0 - A_0}\ln\left(\frac{1}{2}\cdot\frac{A_0}{A_0 - B_0}\right) = T_{12}(A_0, B_0) \tag{55}$$

Combining Equations (54) and (55) the simple cubic Equation (56) has to be solved to get the reaction half-life τ:

$$a\cdot\tau^3 + b\cdot\tau^2 + c\cdot\tau - T_{12}(A_0, B_0) = 0\frac{l}{mol} \tag{56}$$

If Equation (54) is a polynom of higher order than three, Equation (56) only can not be simply solved analytically any more giving an exact formula for τ, which makes a computer calculation necessary.

If a quadratic iteration for $T_{11}(t)$ is chosen (a = 0.l mol^{-1} h^{-3}), τ will be given according to Equation (57):

$$\tau = \frac{1}{2b}\left[-c + \sqrt{c^2 + \frac{8b}{(2B_0 - A_0)}\ln\left(\frac{1}{2}\cdot\frac{A_0}{A_0 - B_0}\right)}\right] \tag{57}$$

In the case of a cubic iteration ($a \neq$ 0.l mol^{-1} h^{-3}), two further terms $T_{13}(a,b,c,A_0,B_0)$ and $T_{14}(a,b,c)$ are introduced in Equation (58):

$$T_{13}(a,b,c,A_0,B_0) = 2b^3 - 9abc - 27a^2 T_{12}(A_0,B_0) \quad \textit{and} \quad T_{14}(a,b,c) = 4(3ac - b^2)^3 \tag{58}$$

If $T_{13}(a,b,c,A_0,B_0) > -T_{14}(a,b,c)$ then according to Equation (59) τ is calculated by:

$$\tau = \frac{1}{3a} \cdot \sqrt[3]{\frac{1}{2}} \cdot \left[-b\sqrt[3]{2} + \sqrt[3]{-T_{13} + \sqrt{T_{13}^2 + T_{14}}} + \sqrt[3]{-T_{13} - \sqrt{T_{13}^2 + T_{14}}} \right] \tag{59}$$

If $T_{13}(a,b,c,A_0,B_0) < -T_{14}(a,b,c)$ then according to Equation (60) τ is the only sensible one (0 h < τ < t_n) of the following possible three solutions with ($\alpha_{1,2} = \pm 60\,° \wedge z_{1,2} = -1) \vee (\alpha_3 = 0\,° \wedge z_3 = 1$):

$$\tau_{1,2,3} = \frac{1}{3a} \cdot \sqrt[6]{\frac{1}{4}} \cdot \left[-b\sqrt[6]{4} + 2z\sqrt[6]{-T_{14}} \cdot \cos\left(\alpha + \frac{1}{3}\arccos\left(-\frac{T_{13}}{\sqrt{-T_{14}}} \right) \right) \right] \tag{60}$$

If using the Equation (60) the relationship $-\pi/2 < (-T_{13})/\mathrm{sqr}(-T_{14}) < \pi/2$ for the argument of the arcus cosine function is not valid and additionally $(-1/9)*(b/a)^2 + (c/3a) < 0$ h^2 holds true, then τ must be calculated by (61) with $p = (-1/9)*(b/a)^2 + (c/(3a))$ and $q = (b^3/(27a^3))-(1/6)*(bc/a^2)-(T_{12}/(2a))$:

$$\tau = -2\sqrt{-p} \cdot \frac{1}{\sin\left(2\arctan\left(\sqrt[3]{\tan\left(\frac{1}{2}\arcsin\left(\frac{\sqrt{-p^3}}{q} \right) \right)} \right) \right)} - \frac{b}{3a} \tag{61}$$

To calculate the **half-life from the iteration parameters *a*, *b* and *c* using the concentration profile of the analyte A**, the term $T_{12}(A_0, B_0)$ is in this case calculated by Equation (62):

$$T_{11}(t=\tau) = \frac{1}{A_0 - B_0} \cdot \ln\left(\frac{2A_0 - B_0}{A_0} \right) = T_{12}(A_0, B_0) \tag{62}$$

Using a quadratic iteration for $T_{11}(t)$ (a = 0.1 mol^{-1} h^{-3}), τ is the only sensible solution of the two possible as calculated by Equation (63):

$$\tau_{1,2} = -\frac{1}{2b}\left(c \pm \sqrt{c^2 + \frac{4b}{A_0 - B_0} \cdot \ln\left(\frac{2A_0 - B_0}{A_0} \right)} \right) \tag{63}$$

Using a cubic iteration ($a \neq$ 0.1 mol^{-1} h^{-3}), Equations (58) to (61) can be used again but after insertion of the term $T_{12}(A_0, B_0)$ as calculated in Equation (62).

© 2013 by the authors; licensee MDPI, Basel, Switzerland. This article is an open access article distributed under the terms and conditions of the Creative Commons Attribution license (http://creativecommons.org/licenses/by/3.0/).
